# Extracellular vesicles and exosomes generated from cystic renal epithelial cells promote cyst growth in autosomal dominant polycystic kidney disease

**DOI:** 10.1038/s41467-021-24799-x

**Published:** 2021-07-27

**Authors:** Hao Ding, Linda Xiaoyan Li, Peter C. Harris, Junwei Yang, Xiaogang Li

**Affiliations:** 1 Department of Internal Medicine, Mayo Clinic, Rochester, MN USA; 2 Department of Biochemistry and Molecular Biology, Mayo Clinic, Rochester, MN USA; 3 Center for Kidney Disease, Second Affiliated Hospital, Nanjing Medical University, Nanjing, China

**Keywords:** Cell biology, Exocytosis, Polycystic kidney disease, Nephrology

## Abstract

Autosomal dominant polycystic kidney disease (ADPKD) is caused by germline mutations of *PKD1* or *PKD2* on one allele and a somatic mutation inactivating the remaining normal allele. However, if and how null ADPKD gene renal epithelial cells affect the biology and function of neighboring cells, including heterozygous renal epithelial cells, fibroblasts and macrophages during cyst initiation and expansion remains unknown. Here we address this question with a “cystic extracellular vesicles/exosomes theory”. We show that cystic cell derived extracellular vesicles and urinary exosomes derived from ADPKD patients promote cyst growth in *Pkd1* mutant kidneys and in 3D cultures. This is achieved by: 1) downregulation of *Pkd1* gene expression and upregulation of specific miRNAs, resulting in the activation of PKD associated signaling pathways in recipient renal epithelial cells and tissues; 2) the activation of fibroblasts; and 3) the induction of cytokine expression and the recruitment of macrophages to increase renal inflammation in cystic kidneys. Inhibition of exosome biogenesis/release with GW4869 significantly delays cyst growth in aggressive and milder ADPKD mouse models, suggesting that targeting exosome secretion has therapeutic potential for ADPKD.

## Abstract (web summary)

Autosomal dominant polycystic kidney disease is characterized by the formation of cysts in the kidney. Here the authors show that cystic extracellular vesicles/exosomes play a critical role in regulating the biology and function of adjacent cells, including renal epithelial cells, fibroblasts and macrophages, and contribute to renal cyst growth.

## Introduction

Autosomal-dominant polycystic kidney disease (ADPKD) is considered the most common monogenic-inherited kidney disease, mainly caused by mutations in either *PKD1* (~85%) or *PKD2* (~15%) with an incidence estimated to be ~1 in 1000^1^. It is estimated that there are >6 million ADPKD patients worldwide, more than half of whom will develop end-stage renal disease and require dialysis or kidney transplantation by age 60 years^2^. The clinical hallmark of ADPKD is progressive cyst formation and marked enlargement of the kidneys, which is caused by sustained expansion of multiple fluid-filled cysts. Cystic progression leads to crowding of adjacent nephrons, which ultimately results in injury to normal-functioning parenchyma and loss of renal function. It is believed that cysts are clonal in nature (derived from a single cell), arising from cells with inherited heterozygous germline mutations in which, (1) a somatic mutation inactivates the remaining normal allele (two-hit model),^3^ or (2) a yet-to-be-identified non genetic factor causing the amount or function of the PKD protein to fall below a critical threshold (threshold model)^4^. Thus, there are two distinct phases in focal cystogenesis in ADPKD: cyst initiation and cyst expansion. However, if and how the PKD-mutated renal epithelial cells in these two phases affect the adjacent cells, such as to lower the amount and function of PKD proteins and to deregulate signaling pathways in these cells to further promote cyst expansion, remain elusive.

A number of different signaling pathways have been associated with cyst formation in ADPKD patients and animal models, including Ca^2+^, cAMP, Wnt/β-catenin, STAT1 and STAT3, ERK and mTOR, TNFα and MIF, and Sirtuin1 and Smyd2, etc^5–14^. It should be noted that very few PKD associated pathways are known to be directly regulated by the PKD1 and/or 2 gene and none of them has been proved to directly regulate the amount and function of the PKD proteins in cystic renal epithelial cells and in other neighboring cells, including ADPKD heterozygous renal epithelial cells, fibroblasts, and immune cells. In addition to direct cell-to-cell contacts influencing the function of adjacent PKD mutant cells, they may affect more distant cells through a secreted factor.

Extracellular vesicles/exosomes have drawn considerable attention as they are implicated in many pathophysiological processes of human diseases^15–17^. Exosomes are small (30–150 nm) vesicles that originate in the endosomal compartment of parent cells^18^. Exosomes have been recognized as important messengers for intercellular communication via transfer of nucleic acids^19^ and specific repertoires of proteins^20^ and lipids^21^ to both adjacent cells and distant cells via the circulation^22–24^. For example, exosomes can transfer inhibitory receptor/ligands and nucleic acids from a tumor to recipient cells^25,26^. Exosomes can protect their cargoes from clearance or damage by complement fixation or macrophages due to their double-layered membrane and nanoscale, thus having long circulation half-life and increasing their biological activity^27^. Blood and urinary exosomes have been proposed to mediate disease and be a potential diagnostic tool in human diseases^28^, including ADPKD^28,29^. However, the basic biological understanding of the exosomes in renal cellular communication and cyst expansion in ADPKD is lacking.

In this work, we provide evidence that cystic epithelial cell-secreted extracellular vesicles/exosomes regulate the biology and function of adjacent cells, including renal epithelial cells, fibroblasts, and macrophages, and contribute to renal cyst formation; inhibition of exosome biogenesis/release delays cyst growth in *Pkd1* mutant mouse kidneys. Our results support a “cystic extracellular vesicles/exosomes theory” in ADPKD, which not only leads to a better understanding of the roles of extracellular vesicles/exosomes in renal cyst formation, but also provides a potential therapeutic strategy for ADPKD.

## Results

### Cyst renal epithelial cell-derived exosomes can be taken up by recipient cells

**Fig. 1:**
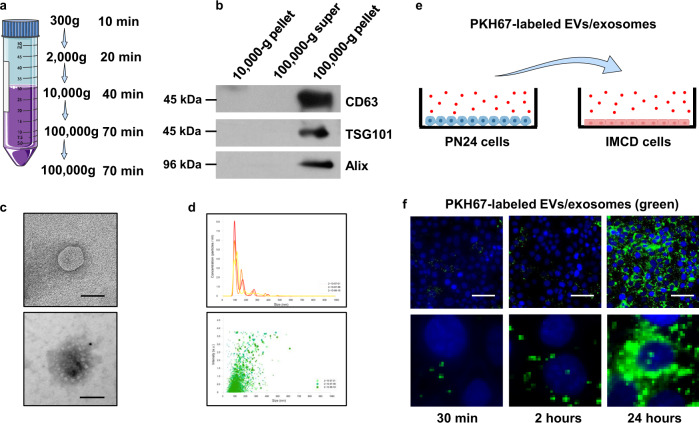
*Pkd1*-null renal epithelial cell-derived EVs/exosomes can be taken by recipient cells. **a** Protocol of the EVs/exosome isolation and purification from FBS-free media. **b** Western blot analysis of the 10,000-g pellet, 100,000-g supernatant, and 100,000-g pellet from FBS-free media by sequential centrifugation. The CD63, TSG101, and Alix proteins, markers of exosomes, were abundant in the 100,000-g pellet isolated from FBS-free media. **c** Transmission electron microscopy (TEM) of EVs/exosomes in FBS-free media (top panel) and Immunogold labeled exosomes (bottom panel). Scale bars, 100 nm. **d** EVs/exosome detection by nanoparticle-tracking analysis (NTA) in FBS-free media. The most often particle diameter is on average of 109.8 nm (top panel), and the distribution histogram showed that the particle diameters were concentrated in the range of 100–110 nm (bottom panel). **e** Schematic of EVs/exosome uptake experiment. Purified PN24 cell-derived EVs/exosomes were labeled with green fluorescent dye, PKH67, and incubated with mouse IMCD3 cells. **f** Mouse IMCD3 cells were incubated with PKH67-labeled (green) EVs/exosomes from PN24 cells for 30 min, 2 h, and 24 h, and fixed for immunofluorescence imaging. Scale bars, 100 μm.

Here we hypothesized that cystic renal epithelial cells might affect neighboring cell function via their secreted extracellular vesicles/exosomes. To test our hypothesis, first, we isolated extracellular vesicles (EVs)/exosomes from the supernatant of cell cultures of postnatal *Pkd1* homozygous PN24 cells (*Pkd1*-null cell EVs/Exosomes) and the same origin postnatal *Pkd1* heterozygous PH2 cells by sequential centrifugation (PH2 cell EVs/exosomes) (Fig. 1a). The presentation of the exosomal marker CD63, Alix and TSG101 in the pellets after ultracentrifugation indicated that those pellets contained exosomes (Fig. 1b). Transmission electron microscopy (TEM) images showed that these EVs had exosome qualities, being typically spherical (Fig. 1c, top panel) and positive for exosomes with immunogold labeling (Fig. 1c bottom panel). Nanoparticle analysis (NTA) revealed that the average diameter of the most common particle is 109.8 nm, and the distribution histogram further showed that the diameter of this particle was concentrated in the range of 100–110 nm (Fig. 1d). The EVs/Exosome particles derived from PN24 cells were increased compared to those derived from PH2 (Supplementary Fig. 1a), and the abundance of EVs/exosome protein and the levels of CD63 in PN24 cell derived EVs/Exosomes were also increased compared to those in PH2 cell-derived EVseExosomes (Supplementary Fig. 1b, c). All of these analyses were performed with the same numbers of cells used for isolation of EVs/exosomes (Supplementary Fig. 1b, c). To test if the *Pkd1*-null cell EVs/exosomes can be taken up by recipient cells, we labeled the EVs/exosomes with a green fluorescent marker, PKH67, which is a membrane dye and has been successfully used to label EVs and exosomes in vitro and in vivo^30^. We then incubated the labeled EVs/exosomes with cultured mouse inner medullary-collecting duct 3 (mIMCD3) cells (Fig. 1e). After removing the EV/exosome-containing media and washing the cells, we found that the labeled *Pkd1*-null cell EVs/exosomes were taken by mIMCD3 cells in a time-dependent manner as examined by immunofluorescence microscopy (Fig. 1f), and enlarged images indicated a cytoplasmic and perinuclear localization of internalized EVs/exosomes (Fig. 1f, *bottom panels*). The membrane association and the entry of PKH67-labeled EV/exosomes (green) in recipient cells was further observed under confocal microscopy (Supplementary Fig. 1d, e).

### Treatment with *Pkd1*-null cell EVs/exosomes repressed the expression of the *Pkd1* gene and activated PKD-associated signaling pathways to promote cystic cell proliferation

**Fig. 2:**
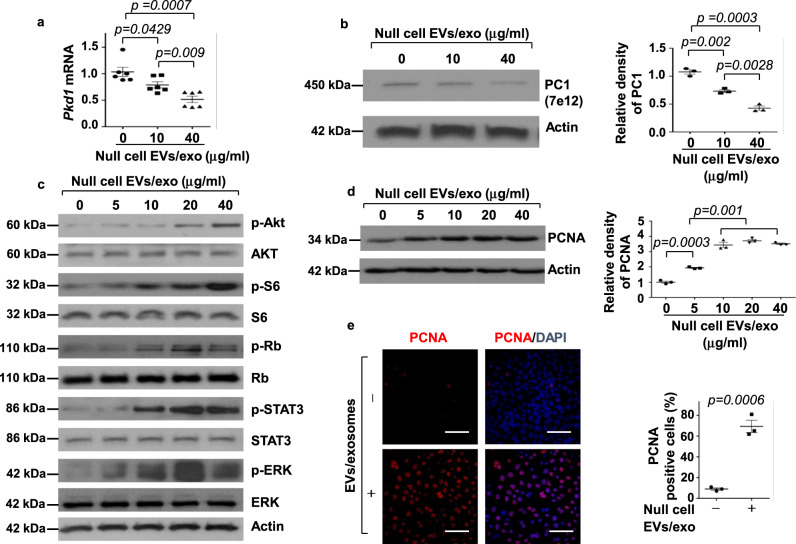
Treatment with *Pkd1*-null cell-derived EVs/exosomes decreased the expression of *Pkd1* gene but induced the activation of PKD-associated signaling pathways to promote cystic cell proliferation. **a** qRT-PCR analysis of *Pkd1* mRNA expression in mIMCD3 cells treated with or without PN24 cell-derived EVs/exosomes. Data were analyzed from five experiments. **b** Western blot analysis of polycystin 1 (PC1) expression from whole-cell lysates of mIMCD3 cells treated with or without PN24 cell-derived EVs/exosomes. Data were analyzed from three experiments. **c** Western blot analysis of the phosphorylation and total proteins of AKT, mTOR, S6, Rb, STAT3, and ERK in mIMCD3 cells treated with or without PN24 cell-derived EVs/exosomes. **d** Western blot analysis of PCNA expression in mIMCD3 cells treated with or without PN24 cell-derived EVs/exosomes. Data were analyzed from three experiments. **e** Immunostaining for PCNA in mIMCD3 cells treated with or without PN24 cell-derived EVs/exosomes (40 μg/ml) for 48 h. Cells were costained with DAPI to visualize the nuclei. Scale bars, 100 μm. Data were analyzed from three experiments. All statistical data are represented as mean ± SEM in **a**, **b**, **d**, and **e**. *P* values by one-way ANOVA followed by Tukey’s post hoc test in **a**, **b**, **d** and by two-tailed unpaired *t*-tests in **e** are indicated.

To test if *Pkd1*-null renal epithelia cell-derived EVs/exosomes affect the biology of recipient cells, we treated mIMCD3 cells with *Pkd1*-null cell-derived EVs/exosomes. We found that treatment with *Pkd1*-null cell EVs/exosomes decreased *Pkd1* mRNA in a dose-dependent manner as examined by quantitative real-time reverse-transcription polymerase chain reaction (qRT-PCR) (Fig. 2a), resulting in the decrease of its gene product, polycystin 1 (PC1) in mIMCD3 cells (Fig. 2b). We also found that treatment with *Pkd1*-null cell EVs/exosomes increased the phosphorylation of AKT, S6, Rb, STAT3, and ERK, but had no effect on the total protein levels of AKT, S6, Rb, STAT3, and ERK (Fig. 2c). In contrast, treatment with *Pkd1* heterozygous PH2 cell derived EVs/exosomes (Supplementary Fig. 2a, b) and mouse IMCD3 cell derived EVs/exosomes (Supplementary Fig. 2c, d) did not affect the expression of *Pkd1* and the phosphorylation of AKT, S6, Rb, STAT3, and ERK in mIMCD3 cells. In addition, we isolated EVs/exosomes from the supernatant of cell cultures of *Pkd1* wild-type mouse embryonic kidney (MEK) cells and *Pkd1-*null MEK cells, and found that the levels of CD63 in *Pkd1-*null MEK cell-derived EVs/exosomes were increased compared to those in *Pkd1* wild-type MEK cell-derived EVs/exosomes (Supplementary Fig. 3a). Treatment with *Pkd1*-null MEK cell EVs/exosomes decreased *Pkd1* mRNA and protein and increased the phosphorylation of AKT, S6, Rb, STAT3, and ERK in mIMCD3 cells (Supplementary Fig. 3b,c), whereas treatment with *Pkd1*-wild-type MEK cell EVs/exosomes did not have those effects (Supplementary Fig. 3d, e). Furthermore, we found that treatment with *Pkd1*-null cell EVs/exosomes increased the expression of the proliferating cell nuclear antigen (PCNA), a marker for cell proliferation when examined by Western blotting (Fig. 2d) and immunofluorescence staining (Fig. 2e and Supplementary Fig. 3f). These results suggested that *Pkd1*-null cell-derived EVs/exosomes could decrease the expression of *Pkd1* mRNA and PC1 in *Pkd1* wild-type renal epithelial cells and induce the activation of PKD-associated signaling pathways to increase cell proliferation.

### Urinary exosomes from ADPKD patients stimulated cultured renal epithelial cell proliferation and promoted cystogenesis in 3D collagen gel cultures

**Fig. 3:**
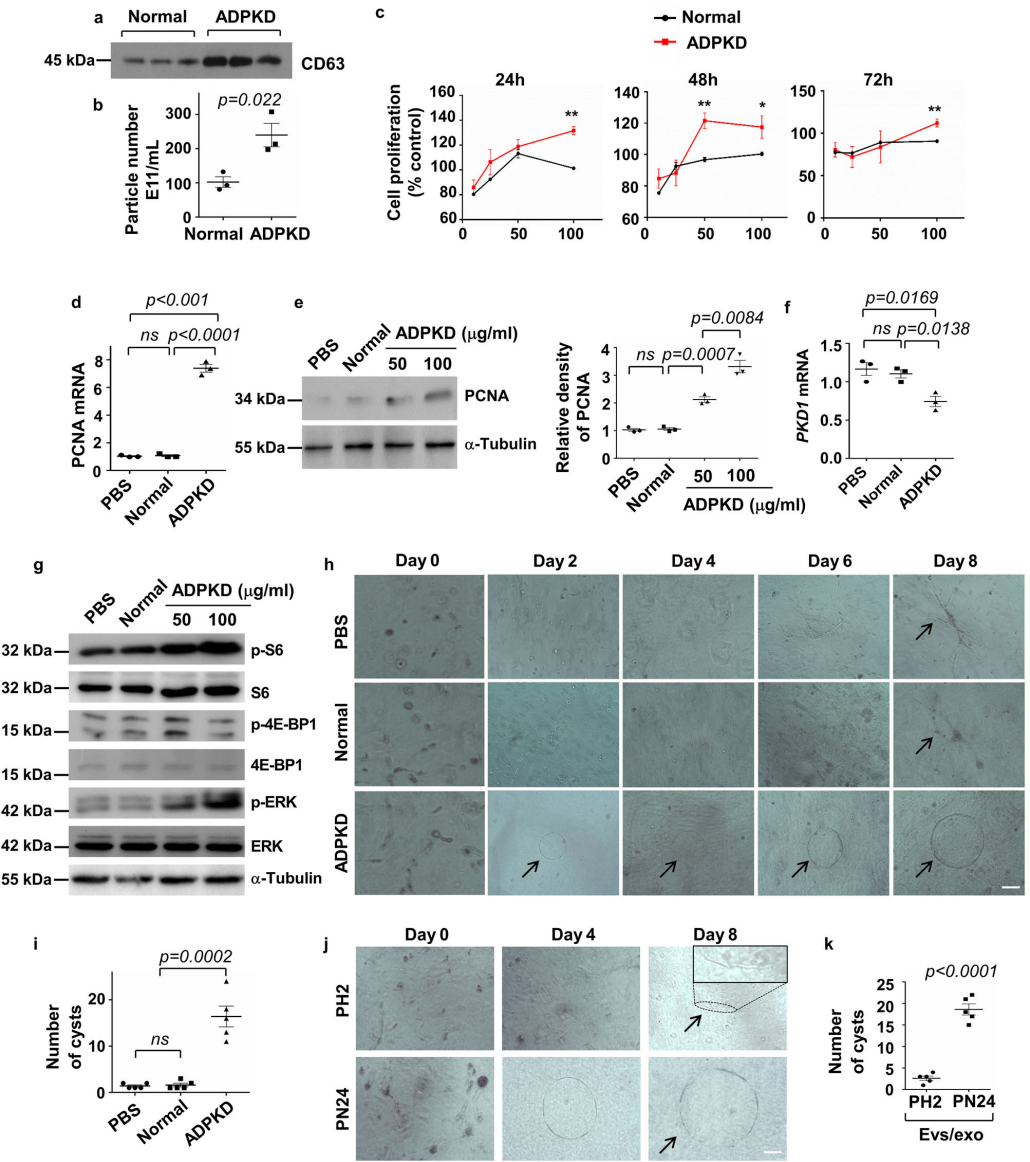
Urinary exosomes from ADPKD patients stimulated cultured tubular cells proliferation and promoted cystogenesis in 3D collagen gel cultures. **a** Western blot analysis of the levels of CD63 in urinary exosomes isolated from 100 ml of urine from ADPKD patients and normal individuals, respectively. **b** The urinary exosome particles derived from 100 ml of urine from ADPKD patients were increased compared to those derived from 100 ml of urine of normal individuals as examined by Nanoparticle analysis (*n* = 3). **c** Treatment with ADPKD urinary exosomes at concentrations of 10, 25, 50, and 100 ug/ml significantly increased mIMCD3 cell proliferation at 24, 48, and 72 h (*n* = 3 technical replicates). qRT-PCR (**d**) and western blot (**e**) analysis of PCNA mRNA and protein levels in mIMCD3 cells treated with PBS or urinary exosomes isolated from normal individuals and ADPKD patients (100 μg/ml) (*n* = 3). The relative PCNA protein levels in renal epithelial cells as standardized to α-Tubulin. qRT-PCR (**f**) analysis of *Pkd1* mRNA expression and western blot (**g**) analysis of the phosphorylation and total proteins of S6, 4EBP-1, and ERK in mIMCD3 cells treated with PBS or urinary exosomes (100 μg/ml) isolated from normal individuals and ADPKD patients (*n* = 3). **h** 3D collagen gel cultures of mIMCD3 cells treated with PBS or urinary exosomes (100 μg/ml) isolated from normal individuals and ADPKD patients and followed for 8 days. Scale bars, 100 μm. **i** Statistic analysis of cyst numbers of 3D cultures in each well at day 8 (*n* = 5 experiments). **j** 3D collagen gel cultures of mIMCD3 cells treated with EVs/exosomes (100 μg/ml) isolated from PH2 and PN24 cells. Scale bars, 100 μm. The tubular structure in 3D culture at day 8 was marked by a dotted border and enlarged in the “inset”. **k** Statistic analysis of cyst numbers of 3D cultures in each well at day 8 (*n* = 5 experiments). All statistical data are represented as mean ± SEM. *P* values by one-way ANOVA followed by Tukey’s post hoc test in **d**, **e**, **f**, and **i** and by two-tailed unpaired *t*-tests in **b** and **k** are indicated.

Urinary exosomes have been proposed to be a potential diagnostic marker in ADPKD^28,29^. However, if urinary exosomes from ADPKD patients can also affect renal epithelial cell biology and function as indicated for cystic cell-derived exosomes is unknown. To test this, we first isolated urinary exosomes from ADPKD patients and healthy individuals (control) with sequential ultracentrifugation (see “Methods”). We found that the levels of CD63 in urinary exosomes isolated from ADPKD patients were increased compared to those in urinary exosomes isolated from healthy individuals as examined by Western blot analysis (Fig. 3a). The number of exosomal particles was also increased in urines from ADPKD patients compared to the healthy individuals as examined by nanoparticle analysis (Fig. 3b). These results suggest that exosomes were increased in urines from ADPKD patients compared to the health individuals. We then found that treatment with ADPKD urinary exosomes at concentrations of 50 and 100 ug/ml significantly increased mIMCD3 cell proliferation compared to those cells treated with urinary exosomes isolated from normal individuals at 24, 48, and 72 h when examined with the MTT (3-(4,5-dimethylthiazol-2-yl)-2,5-diphenyltetrazolium bromide) assay (Fig. 3c). Treatment with ADPKD urinary exosomes also increased mRNA and protein levels of PCNA as examined by qRT-PCR (Fig. 3d) and Western blot analysis (Fig. 3e). We further found that treatment with ADPKD urinary exosomes decreased the expression of *Pkd1* mRNA (Fig. 3f) but increased the phosphorylation of ERK, 4EBP, and S6 with no effect on the total protein levels of ERK, 4EBP, and S6 in IMCD3 cells compared to those cells treated with PBS (control) and normal urinary exosomes (Fig. 3g). These results suggested that ADPKD urinary exosomes contains critical pathological factors, similar to cystic cell-derived exosomes, to regulate the expression of the *Pkd1* gene, activate PKD-associated signaling pathways, and increase renal epithelial cell proliferation. In addition, we found that mIMCD3 cells treated with PBS and normal urinary exosomes developed tubule-like structures in 3D collagen gels within 2 days, that were maintained up to 8 days. In contrast, mIMCD3 cells treated with ADPKD urinary exosomes developed cyst-like structures in collagen gels within 2 days which continued growing progressively up to day 8 (Fig. 3h, i). Furthermore, we found that mouse IMCD3 cells treated with PH2 cell EVs/exosomes resulted in tubule-like structures in 3D collagen gels, whereas those cells treated with PN24 cell EVs/exosomes developed cyst-like structures in collagen gels (Fig. 3j, k). These results suggest that ADPKD urinary exosomes have similar characteristics compared to cystic cell-derived EVs/exosomes.

### Treatment with *Pkd1*-null cell-derived EVs/exosomes promoted cyst growth in vivo

Given the robust evidence of altered cystogenic characteristics of cystic cell-derived EVs/exosomes and ADPKD urinary exosomes in vitro, we investigated if treatment with *Pkd1*-null cell EVs/exosomes promotes cyst growth in vivo. The route(s) of administration of exosomes to mice via intravenous injection has been reported^31^. However, before administration of EVs/exosomes to *Pkd1*^*RC/RC*^ mice, a well-characterized animal model for ADPKD^32^, we first evaluated if the PKH67-labeled EVs/exosomes could be distributed throughout kidneys by intravenous (IV) injections. We treated *Pkd1*^*RC/RC*^ mice with PKH67-labeled exosomes or PBS by intravenous injection for 24 h, and kidneys were harvested and imaged by an in vivo imaging system (IVIS), leading optical imaging technology to monitor disease progression, cell trafficking, and gene expression patterns in living animals^33^. We observed strong fluorescent signals from PKH67-labeled EVs/exosomes derived from *Pkd1*-null cells (Supplementary Fig. 4a, *middle panel*) and PH2 cells (Supplementary Fig. 4a, *right panel*) in the kidneys from these mice, suggesting that EVs/exosomes could enter kidneys with these treatment methods, as previously described^34^.

**Fig. 4:**
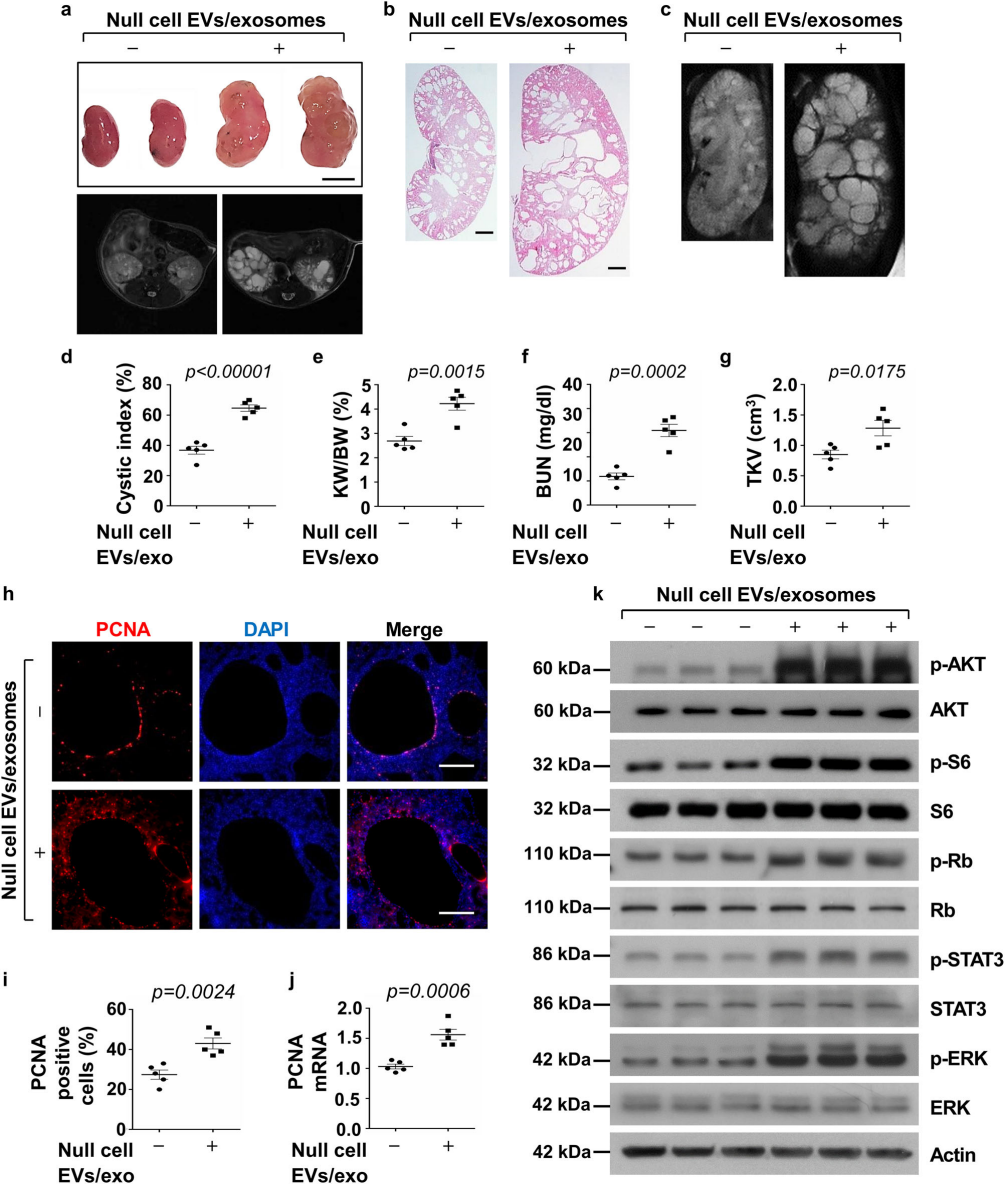
Treatment with *Pkd1*-null cell-derived EVs/exosomes promoted cyst growth in vivo. **a** Images of kidneys (top panel) and axial MRI (bottom panel) from *Pkd1*^*RC/RC*^ mice treated with or without cystic cell-derived EVs/exosomes. Scale bars, 5 mm. **b** Histological examination of kidneys from *Pkd1*^*RC/RC*^ mice treated with or without cystic cell-derived EVs/exosomes. Scale bars, 1 mm. **c** MRI images from *Pkd1*^*RC/RC*^ mice treated with or without cystic cell-derived EVs/exosomes. Scale bars, 1 mm. **d** Treatment with cystic cell-derived EVs/exosomes increased cyst index in kidneys from *Pkd1*^*RC/RC*^ mice treated with cystic exosomes (*n* = 5) compared to that in control mice treated with PBS (*n* = 5). Treatment with cystic exosomes increased KW/BW ratios (**e**) and BUN levels (**f**) in *Pkd1*^*RC/RC*^ mice compared to those in control mice treated with PBS. **g** Total kidney volume (TKV) was calculated by MRI scan in each group. **h** Treatment with cystic cell EVs/exosomes increased cyst-lining epithelial and interstitial cell proliferation in kidneys from *Pkd1*^*RC/RC*^ mice as detected with PCNA staining. Scale bars, 100 μm. **i** The percentage of PCNA-positive cells was calculated from an average of 1000 nuclei per mouse kidney section. **j** qRT-PCR analysis of PCNA mRNA expression in kidneys from *Pkd1*^*RC/RC*^ mice treated with cystic cell EVs/exosomes or PBS. **k** Western blot analysis of the phosphorylation and total proteins of AKT, mTOR, S6, Rb, STAT3 and ERK in kidneys from *Pkd1*^*RC/RC*^ mice treated with cystic cell EVs/exosomes or PBS. All statistical data are represented as mean ± SEM, and *p* values are calculated by unpaired Student’s *t*-test.

For the in vivo study, we treated 1-month old *Pkd1*^*RC/RC*^ mouse in 129 S/6 background with *Pkd1*-null cell EVs/exosomes (200 ug in sterile PBS) and PBS (control) by intravenous injection three times per week up to 3 months, when the kidneys were harvested (Supplementary Fig. 4a, b). We found that administration of *Pkd1*-null cell EVs/exosomes promoted cyst growth in kidneys from *Pkd1*^*RC/RC*^ characterized by enlarged kidney size (Fig. 4a–c) and increased cystic index (Fig. 4d) and kidney weight/body weight (KW/BW) ratios (Fig. 4e) compared to those in kidneys from *Pkd1*^*RC/RC*^ mice treated with PBS, PH2 cell-derived EVs/exosomes (Supplementary Fig. 5a-e) and mIMCD3 cell-derived EVs/exosomes (Supplementary Fig. 6a-g), respectively. Treatment with *Pkd1*-null cell EVs/exosomes aggravated renal function in *Pkd1*^*RC/RC*^ mice as indicated by the increase of blood urea nitrogen (BUN) levels in these mice compared to the control mice (Fig. 4f). Total kidney volume (TKV), which was assessed with magnetic resonance imaging (MRI), was also significantly increased in *Pkd1*^*RC/RC*^ treated with *Pkd1*-null cell EVs/exosomes compared to that in the control mice treated with PBS (Fig. 4g). We further found that treatment with *Pkd1*-null cell EVs/exosomes not only increased cyst lining epithelial cell proliferation but also the surrounding interstitial cell proliferation in *Pkd1*^*RC/RC*^ kidneys compared to PBS-treated *Pkd1*^*RC/RC*^ kidneys, as analyzed by PCNA staining and qRT-PCR analysis (Fig. 4h–j) and Ki67 staining (Supplementary Fig. 7a), suggesting that *Pkd1*-null cell EVs/exosomes might affect the biology of neighboring cells, such as fibroblasts and macrophages. Consistent with the downregulation of *Pkd1* expression in mIMCD3 cells treated with cystic cell EVs/exosomes, we also found that treatment with *Pkd1*-null cell EVs/exosomes decreased the levels of *Pkd1* mRNA in *Pkd1*^*RC/RC*^ kidneys compared to the controls (Supplementary Fig. 7b). In addition, we found that administration of cystic cell EVs/exosomes increased the phosphorylation of AKT, S6, Rb, STAT3, and ERK but had no effect on the protein levels of AKT, S6, Rb, STAT3, and ERK in *Pkd1*^*RC/RC*^ kidneys compared to those in the control kidneys (Fig. 4k).

In addition, to address whether treatment with *Pkd1*-null cell EVs/exosomes could promote cyst formation in normal mice, we treated *Pkd1*^*flox/+*^*:Pkhd1-Cre* mice with *Pkd1*-null cell EVs/exosomes or PBS from postnatal day 7 (P7) to 3-month old. We found that *Pkd1*-null cell EVs/exosomes could induce tubular dilation and small cyst formation as examined with MRI image and H&E staining (Supplementary Fig. 8a), and renal fibrosis as examined by picrosirius red and fibronectin staining (Supplementary Fig. 8b) in kidneys of *Pkd1*^*flox/+*^*:Pkhd1-Cre* mice but not those in kidneys from PBS-treated control mice. In addition, we found that treatment with *Pkd1*-null cells EVs/exosomes decreases the expression of *Pkd1* mRNA level in kidneys of *Pkd1*^*flox/+*^*:Pkhd1-Cre* mice (Supplementary Fig. 8c). In addition, treatment with *Pkd1*-null cell EVs/exosomes not only increased cyst lining epithelial cell proliferation but also the surrounding interstitial cell proliferation (Supplementary Fig. 8d) as in *Pkd1*^*RC/RC*^ mice. However, the milder phenotypes observed in *Pkd1*-null cell EVs/exosome-treated *Pkd1*^*flox/+*^*:Pkhd1-Cre* mouse kidneys did not result in the significant changes of cystic index and KW/BW ratios compared to those in control mice. Interestingly, we observed that glomeruli were enlarged and glomerular capillary loops were developing cystic-like structures in kidneys from cystic cell EVs/exosome-treated *Pkd1*^*flox/+*^*:Pkhd1-Cre* mice and *Pkd1*^*RC/RC*^ mice (Supplementary Fig. 8e), suggesting that *Pkd1*-null EVs/exosomes might also affect vascular endothelial cells.

### Treatment with *Pkd1*-null cell EVs/exosomes increased fibrosis in *Pkd1*^*RC/RC*^ kidneys and enhanced the activation of NRK-49F cells

**Fig. 5:**
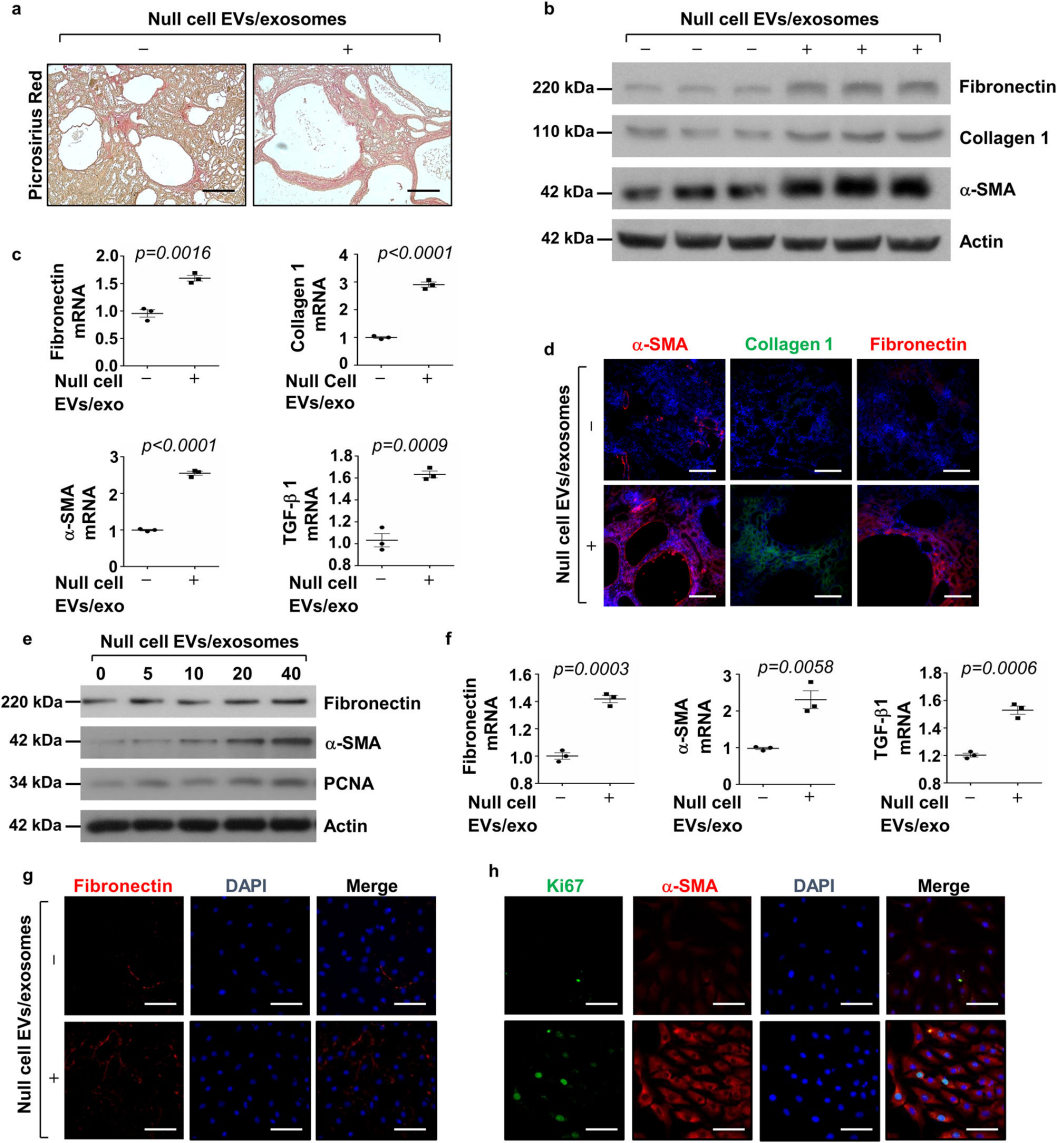
Treatment with *Pkd1*-null cell EVs/exosomes increased fibrosis in *Pkd1*^*RC/RC*^ kidneys and induced the activation of NRK-49F cells. **a** Picrosirius red staining revealed that renal fibrosis was increased in kidneys of *Pkd1*^*RC/RC*^ mice treated with cystic cell EVs/exosomes compared to that in kidneys of *Pkd1*^*RC/RC*^ mice treated with PBS. Scale bars, 50 μm. **b** Western blot analysis of fibronectin, collagen 1, and α-SMA, expression in kidneys from *Pkd1*^*RC/RC*^ mice treated with cystic cell EVs/exosomes or PBS. **c** qRT-PCR analysis of fibronectin, collagen 1, α-SMA and TGF-β mRNA expression in kidneys from *Pkd1*^*RC/RC*^ mice treated with cystic cell EVs/exosomes or PBS. All data were analyzed from three experiments. **d** Immunostaining of fibronectin, collagen 1, and α-SMA expression in kidneys from *Pkd1*^*RC/RC*^ mice treated with cystic cell EVs/exosomes or PBS. **e** Western blot analysis of fibronectin, α-SMA and PCNA expression from whole-cell lysates of rat kidney fibroblasts (NRK-49F) treated with or without cystic cell EVs/exosomes. **f** qRT-PCR analysis of fibronectin, α-SMA and TGF-β mRNA expression from whole cell lysates of rat kidney fibroblasts (NRK-49F) treated with or without cystic cell EVs/exosomes. All data were analyzed from three experiments. Immunostaining of fibronectin (**g**), Ki67, and α-SMA (**h**) in NRK-49F cells treated with cystic cell EVs/exosomes or PBS. All statistical data are represented as mean ± SEM, and *p*-values are calculated by unpaired Student’s *t*-test.

In ADPKD, cyst progression results in not o ^35^. We found that treatment with *Pkd1*-null cell EVs/exosomes increased extracellular matrix production in the kidney interstitium of *Pkd1*^*RC/RC*^ mice compared to that in PBS-treated control mice as examined with picrosirius red staining (Fig. 5a). We further found that this treatment increased the expression of fibronectin, collagen 1, and α-SMA in kidneys from *Pkd1*^*RC/RC*^ mice compared to those in kidneys from *Pkd1*^*RC/RC*^ mice treated with PBS as examined by Western blotting (Fig. 5b) and qRT-PCR analysis (Fig. 5c) as well as immunofluorescence staining (Fig. 5d). These results suggested that EVs/exosomes from *Pkd1*-null renal epithelial cells could activate fibroblasts and increase abnormal deposition of the extracellular matrix (ECM), resulting in renal fibrosis. To further support this notion, we found that treatment with *Pkd1*-null cell EVs/exosomes increased the mRNA and protein levels of fibronectin, α-SMA, TGF-β, as well as PCNA in rat kidney fibroblasts (NRK-49F) in a dose-dependent manner (Fig. 5e–h).

### *Pkd1*-null renal epithelial cells that secreted EVs/exosomes induced the expression of miRNAs that are involved in cyst pathogenesis

**Fig. 6:**
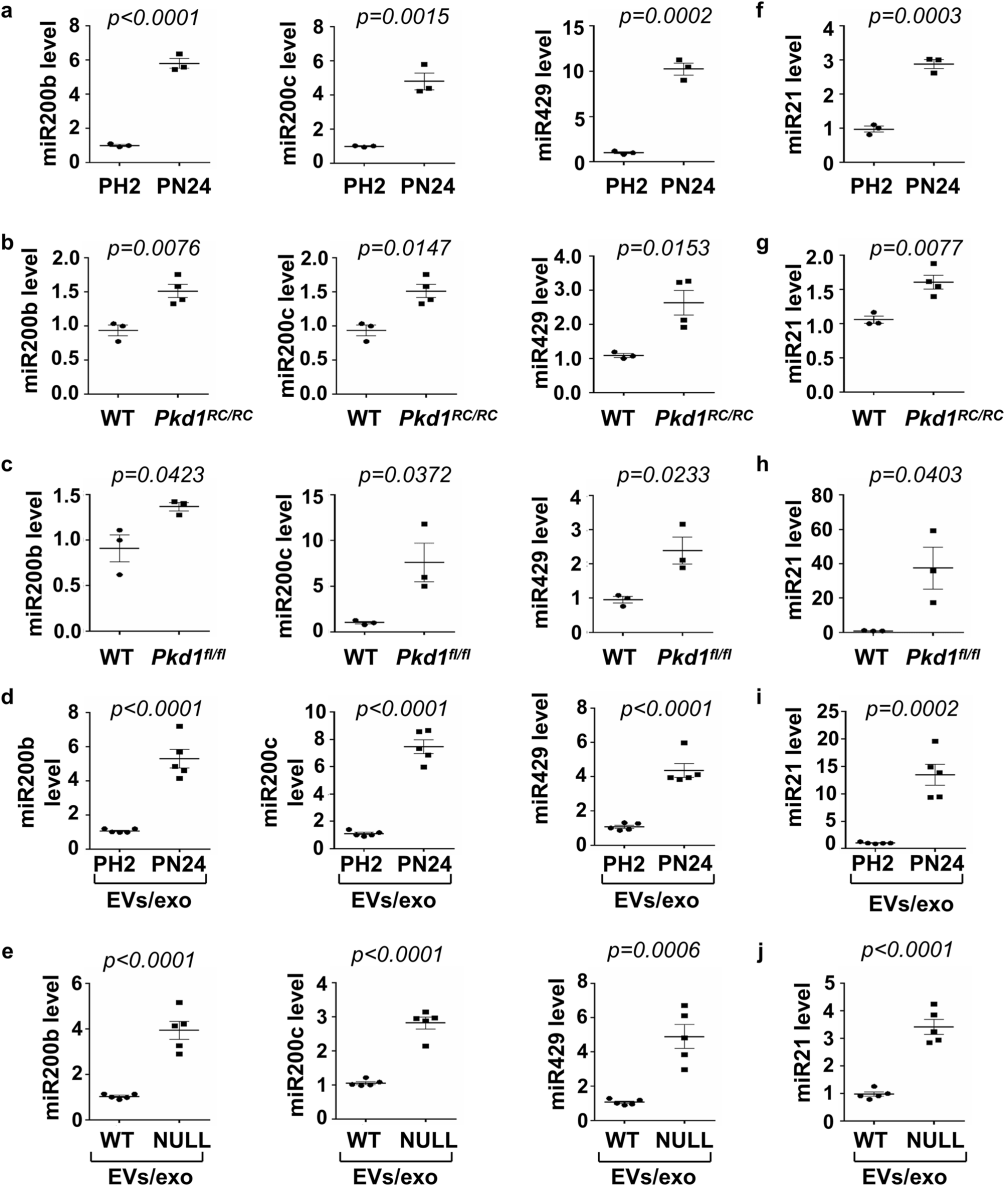
The expression of miR200s and miR21 was increased in cystic renal epithelial cells and tissues. **a** qRT-PCR analysis of the expression of miR200b, miR200c, and miR429 in PH2 and PN24 cells. All data were analyzed from three experiments. **b** qRT-PCR analysis of the expression of miR200b, miR200c, and miR429 in kidneys of WT and *Pkd1*^*RC/RC*^ mice. All data were analyzed from 4 experiments. **c** qRT-PCR analysis of the expression of miR200b, miR200c and miR429 in kidneys of WT and *Pkd1*^*flox/flox*^:*Pkhd1-Cre* mice. All data were analyzed from three experiments. **d** qRT-PCR analysis of the expression of miR200b, miR200c and miR429 in 1 mg of EVs/exosomes derived from PH2 and PN24 cells. All data were analyzed from five experiments. **e** qRT-PCR analysis of the expression of miR200b, miR200c, and miR429 in 1 mg EVs/exosomes derived from *Pkd1* wild-type and null MEK cells. All data were analyzed from five experiments. qRT-PCR analysis of the expression of miR21 in PH2 and PN24 cells (**f**) and in kidneys of WT and *Pkd1*^*RC/RC*^ mice (**g**) as well as in kidneys of WT and *Pkd1*^*flox/flox*^:*Pkhd1-Cre* mice (**h**). **i** qRT-PCR analysis of the expression of miR21 in 1 mg of EVs/exosomes derived from the PH2 and PN24 cells. All data were analyzed from 3 to 5 experiments. **j** qRT-PCR analysis of the expression of miR21 in 1 mg of EVs/exosomes derived from *Pkd1* wild-type and null MEK cells. All data were analyzed from five experiments. All statistical data are represented as mean ± SEM, and p-values are calculated by unpaired Student’s *t*-test.

EVs/exosomes deliver a diverse array of biomolecules including microRNAs (miRNAs)^36^. MicroRNAs are aberrantly expressed in cystic kidneys and are thought to regulate key aspects of cyst pathogenesis^37^. Bioinformatics comparison of five different databases predicts that miR-200 family members directly bind to *Pkd1* 3’-UTR and inhibit its translation (Supplementary Fig. 9). We found that the expression of miR200 family, including miR-200b, miR-200c, and miR-429, was increased in postnatal *Pkd1* homozygous PN24 cells compared to *Pkd1* heterozygous PH2 cells (Fig. 6a) as well as in kidneys from *Pkd1*^*RC/RC*^ (Fig. 6b) and *Pkd1*^*flox/flox*^*:Pkhd1-Cre* mice (Fig. 6c) compared to that in kidneys from WT mice as examined by qRT-PCR analysis. The upregulated miRNAs in *Pkd1*-null renal epithelial cells increased their secretion in *Pkd1*-null cell-derived EVs/exosomes compared to that in EVs/exosomes derived from PH2 and *Pkd1* wild-type MEK cells (Fig. 6d, f), which should facilitate their effects on recipient cells as described above. To support this notion, we found that treatment with *Pkd1*-null cell EVs/exosomes induced the expression of miR200s in mouse IMCD3 cell (Supplementary Fig. 10a). Recent studies have demonstrated that elevated expression of miR-21 plays a vital role in the development of fibrosis by promoting the proliferation of interstitial fibroblasts and increasing the abnormal deposition of the ECM^38^. We found the expression of miR21 was also increased in PN24 cells and kidneys from *Pkd1* mutant mice compared to that in PH2 cells and kidneys from WT mice (Fig. 6f–h). The levels of miR21 were also increased in *Pkd1*-null cell-derived EVs/exosomes compared to that in EVs/exosomes derived from PH2 and *Pkd1* wild-type MEK cells (Fig. 6i, j). Consistently with miR200s, treatment with *Pkd1*-null cell EVs/exosomes also induced the expression of miR21 in mIMCD3 cells (Supplementary Fig. 10b). The induction of miR200s and miR21 by cystic cell EVs/exosomes may modulate *Pkd1* gene transcription. To support this, we found that treatment with mimics of miR200b/c, miR429 but not miR21decreased the expression of *Pkd1* mRNA in IMCD3 cells (Supplementary Fig. 10c), which should increase the activation of PKD-associated pathways in recipient cells and the activation of fibroblasts. Taken together, these data revealed that cyst renal epithelial cell-derived EVs/exosomes could induce the expression of miRNA-related cyst growth in recipient cells to form a positive feedback loop between miRNAs in cystic cells and miRNAs in EVs/exosomes, as well as miRNAs in neighboring cells, which should be one of the mechanisms for cystic cell-derived EVs/exosomes to promote cyst growth and renal fibrosis in cystic kidneys.

### The expression of genes associated with EVs/exosome biogenesis upregulated in *Pkd1* mutant renal epithelial cells and tissues

**Fig. 7:**
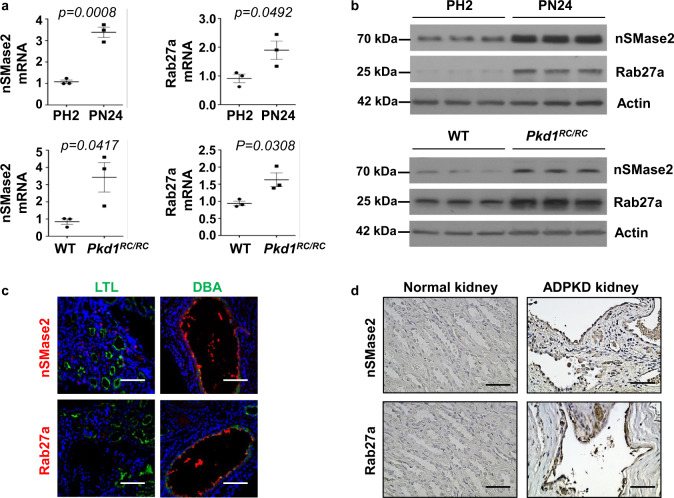
The expression of exosome biogenesis/release-associated genes was upregulated in *Pkd1* mutant renal epithelial cells and tissues. **a** qRT-PCR analysis of the expression of nSMase2 and Rab27a mRNA in PH2 and PN24 cells (top panel) and in kidneys from WT and *Pkd1*^*RC/RC*^ mice (bottom panel). *n* = 3 independent experiments. All statistical data are represented as mean ± SEM, and p-values are calculated by unpaired Student’s *t*-test. **b** Western blot analysis of nSMase2 and Rab27a expression from whole-cell lysates of PH2 cells and PN24 cells (top panel) and in kidneys from WT and *Pkd1*^*RC/RC*^ mice (bottom panel). **c** Immunofluorescence staining indicated that the expression of nSMase2 and Rab27a was increased in cyst lining epithelial cells in kidneys from *Pkd1*^*RC/RC*^ mice, which were costained with DBA (Dilichos biflorus agglutinin) but not LTL (Lotus Tetragonolobus lectin). Scale bars, 100 μm. *n* = 3 independent experiments. **d** Immunohistochemistry analysis indicated that nSMase2 and Rab27a expression was increased in cyst-lining epithelial cells in human ADPKD kidneys but not that in normal human kidneys. Scale bars, 50 μm. *n* = 3 independent experiments.

The results that treatment with cystic cell EVs/exosomes significantly promoted cyst growth in *Pkd1*^*RC/RC*^ mice supported a role of cystic cell exosomes in vivo. However, if exosome biogenesis/release is changed in cystic renal epithelial cells and tissues is unknown. There are key enzymes that regulate exosome biogenesis/release, including neutral sphingomyelinase 2 (nSMase 2)^39^ which promotes budding of intravesicular vesicles, and Ras-related protein 27a (Rab27a), which is involved in the fusion of the MVB to the plasma membrane^40,41^. We found that the expression of NSMASE2 and RAB27A was upregulated in postnatal *Pkd1* homozygous PN24 cells compared to that in postnatal *Pkd1* heterozygous PH2 cells as examined by qRT-PCR (Fig. 7a) and Western blot analysis (Fig. 7b). We also found that the expression of NSMASE2 and RAB27A mRNA and protein was upregulated in kidneys from 3-month-old *Pkd1*^*RC/RC*^ mice, compared to that in kidneys from age matched *Pkd1* wild-type mice (Figs. 7a, b). NSMASE2 and RAB27A were mainly expressed in cyst-lining epithelial cells in kidneys from *Pkd1*^*RC/RC*^ mice and ADPKD patients as examined by immunofluorescence (Fig. 7c) and immunohistochemistry staining (Fig. 7d), respectively. The upregulation of these two enzymes in cystic kidneys and particularly in cyst-lining epithelial cells suggested that the EVs and exosome secretion from these cells should be increased in cystic kidneys. To examine whether treatment with GW4869 decreases the secretion of EVs/exosomes, we treated PN24 cells with GW4869 and then isolated EVs/exosomes from the supernatant of cell cultures. We found that treatment with GW4869 decreased EVs/exosomes particles derived from PN24 cells compared to those derived from control cells (Supplementary Fig. 11a), and the levels of CD63 and miR200s in GW4869-treated cell-derived EVs/Exosomes were also decreased compared to those in control cell-derived EVs/exosomes. All of these analyses were performed with the same numbers of GW4869-treated and control cells (Supplementary Fig. 11b, c).

### Treatment with an inhibitor, GW4869, of exosome generation delayed cyst growth and alleviated renal fibrosis in *Pkd1* mutant kidneys

**Fig. 8:**
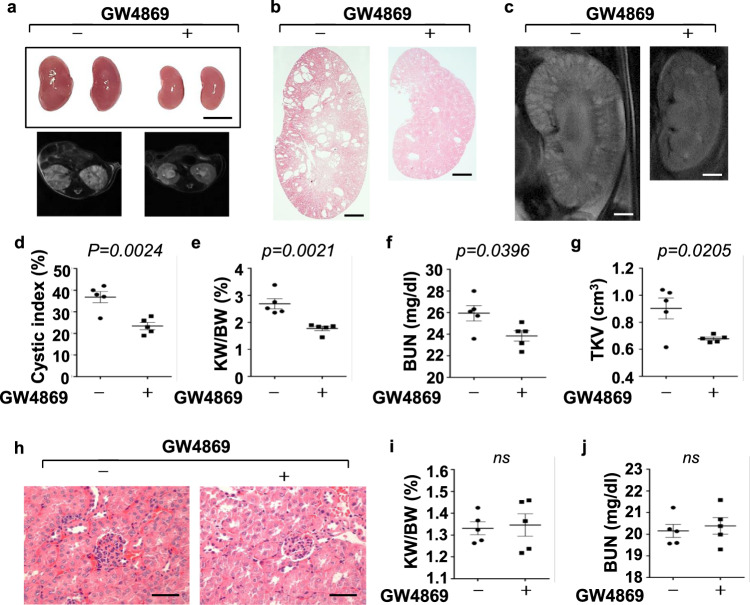
Inhibition of exosome secretion with inhibitor, GW4869, delayed cyst growth in *Pkd1*^*RC/RC*^ mice. **a** Images of kidneys (top panel) and axial MRI images (bottom panel) from *Pkd1*^*RC/RC*^ mice treated with or without GW4869. Scale bars, 5 mm. **b** Histological examination of kidneys from *Pkd1*^*RC/RC*^ mice treated with or without GW4869. Scale bars, 1 mm. **c** MRI images from *Pkd1*^*RC/RC*^ mice treated with or without GW4869. Scale bars, 1 mm. **d** Treatment with GW4869 deceased cystic index in kidneys from *Pkd1*^*RC/RC*^ mice (*n* = 5) compared to that in kidneys from control mice treated with DMSO (*n* = 5). Treatment with GW4869 decreased KW/BW ratios (**e**) and BUN levels (**f**) in *Pkd1*^*RC/RC*^ mice compared to those in kidneys from control mice treated with DMSO. **g** Total kidney volume (TKV) was calculated by MRI scan in each group. **h** Histological examination of kidneys from wild-type mice treated with GW4869 (*n* = 5). Scale bars, 1 mm. Treatment with GW4869 did not affect KW/BW ratios (**i**) and BUN levels (**j**) in GW4869-treated wild-type mice compared to the controls. All statistical data are represented as mean ± SEM, and *p* values are calculated by unpaired Student’s *t*-test.

**Fig. 9:**
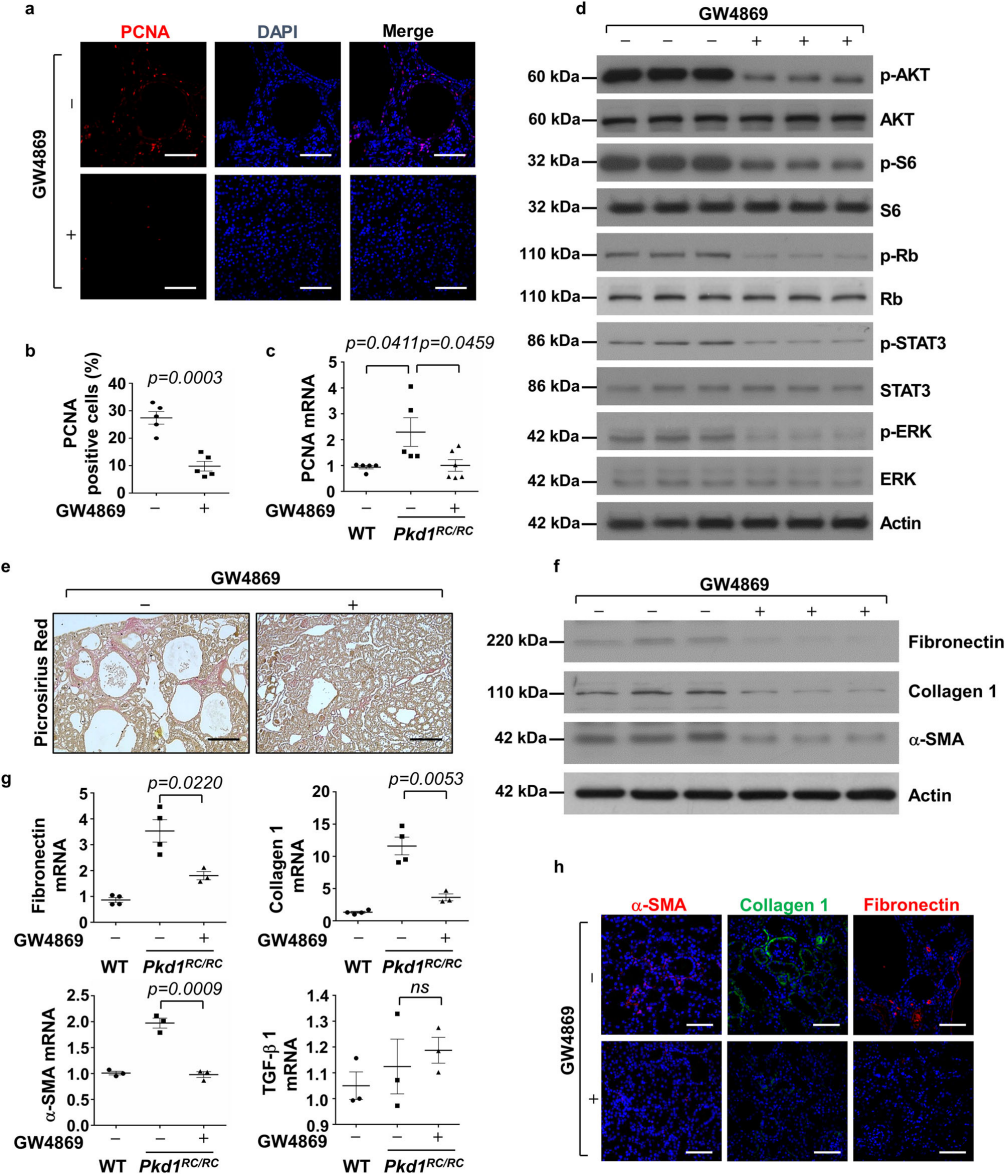
Treatment with GW4869 decreased cell proliferation and renal fibrosis in *Pkd1* mutant kidneys. **a** Treatment with GW4869 reduced cyst-lining and interstitial cell proliferation in kidneys from *Pkd1*^*RC/RC*^ mice as detected with PCNA staining. (*n* = 5). Scale bars, 100 μm. **b** The percentage of PCNA-positive cells was calculated from an average of 1000 nuclei per mouse kidney section. **c** qRT-PCR analysis of the expression of PCNA mRNA in kidneys from *Pkd1*^*RC/RC*^ mice treated with GW4869 (+) or DMSO (−). **d** Western blot analysis of the phosphorylation and total proteins of AKT, mTOR, S6, Rb, STAT3, and ERK in kidneys from *Pkd1*^*RC/RC*^ mice treated with GW4869 (+) or DMSO (−). **e** Picrosirius red staining revealed that renal fibrosis was decreased in kidneys of *Pkd1*^*RC/RC*^ mice treated with GW4869 compared to that in kidneys of *Pkd1*^*RC/RC*^ mice treated with DMSO. Scale bars, 50 μm. **f** Western blot analysis of fibronectin, collagen 1 and α-SMA expression in kidneys from *Pkd1*^*RC/RC*^ mice treated with GW4869 (+) or DMSO (−). **g** qRT-PCR analysis of the expression of fibronectin, collagen 1, α-SMA and TGF-β mRNAs in kidneys from WT and *Pkd1*^*RC/RC*^ mice treated with GW4869 or DMSO. All data were analyzed from 3 to 5 experiments. **h** Immunostaining of fibronectin, collagen 1, and α-SMA, in kidneys from *Pkd1*^*RC/RC*^ mice treated with GW4869 (+) or DMSO (−). All statistical data are represented as mean ± SEM. *P* values by one-way ANOVA followed by Tukey’s post hoc test in **c**, **g** and by two-tailed unpaired *t*-tests in **b** are indicated.

We next tested if inhibition of exosome secretion with inhibitor, GW4869, delayed cyst growth in vivo. GW4869, a neutral sphingomyelinase inhibitor, is a commonly used pharmacological agent to block exosome biogenesis/release^39,42^, with evidence that it can also inhibit exosome release in HEK293 cells^43^. First, we examined if treatment with GW4869 could delay cyst growth in *Pkd1*^*RC/RC*^ mice by treating with GW4869 (5 μg/g dissolved in DMSO) or DMSO (0.005%) by daily intraperitoneal injection from 1 to 3 months. We found that administration of GW4869 slowed cyst growth as seen by decreased cyst index, KW/BW ratio, and BUN level in 3-month kidneys from *Pkd1*^*RC/R*C^ mice compared to those in age-matched kidneys from DMSO-injected *Pkd1*^*RC/RC*^ mice (*n* = 5) (Fig. 8a–f). TKVs of 3-month old *Pkd1*^*RC/RC*^ mice treated with GW4869 were also significantly smaller than the control mice (Fig. 8g). Administration of GW4869 by daily intraperitoneal injection to wild-type mice from 1 to 3 months did not affect the growth of those mice as seen of no effects on KW/BW ratio and BUN levels in those mice (Fig. 8h–j). In addition, treatment with GW4869 decreased cyst-lining epithelial and surrounding cell proliferation as examined by PCNA staining (Fig. 9a, b) and the expression of PCNA as examined by qRT-PCR (Fig. 9c). Administration of GW4869 also decreased the phosphorylation of AKT, S6, Rb, STAT3, and ERK but not the levels of total AKT, S6, Rb, STAT3, and ERK proteins in kidneys of *Pkd1*^*RC/RC*^ mice compared to the controls (Fig. 9d). Furthermore, we found that GW4869 treatment decreased extracellular matrix deposition and the expression of fibronectin, collagen 1 and α-SMA in *Pkd1*^*RC/RC*^ mice kidneys compared to controls, as examined with picrosirius red staining (Fig. 9e), Western blotting (Fig. 9f), qRT-PCR (Fig. 9g) and immunostaining (Fig. 9h), respectively. These data indicated that inhibition of exosome generation also suppressed renal fibrosis. Interestingly, we found that treatment with GW4869 decreased the expression of miR200s and miR21 in kidneys from *Pkd1*^*RC/RC*^ mice compared to controls (Supplementary Fig. 12a, b).

To extend the translational significance of our in vivo findings to an early-stage ADPKD mouse model, we also administrated GW4869 to *Pkd1*^*flox/flox*^:*Pkhd1-Cre* mice, an aggressive ADPKD mouse model, in which the Cre is driven by the *Pkhd1* promoter that results in cyst formation by postnatal day 9 or 10^44^. We found that administration of GW4869 (5 g/g) (*n* = 5) delayed cyst growth (Supplementary Fig. 13a, b) as seen by the decrease of cyst index, KW/BW ratio, and BUN level (Supplementary Fig. 13c–e), and cystic epithelial cell proliferation (Supplementary Fig. 13f) in kidneys from *Pkd1*^*flox/flox*^: *Pkhd1-Cre* mice compared to controls. We also found that treatment with GW4869 decreased the expression of miR200s and miR21 in kidneys from *Pkd1*^*flox/flox*^:*Pkhd1-Cre* mice compared to control-treated mice (Supplementary Fig. 12c, d). In both mouse models, no gender difference was observed for the effect of GW4869 on delaying cyst growth. These results suggested a beneficial effect of inhibition of exosome secretion in ADPKD models.

### Treatment with *Pkd1*-null cell EVs/exosomes and GW4869 affects the expression of cytokines and the recruitment of macrophages in cystic kidneys

Interstitial inflammation, which has been consistently reported in human and animal models of PKD, also regulates cyst growth and is associated with functional impairment. Cytokines, including TNF-α, IL-6, and MCP-1, which are involved in renal inflammation in cystic kidneys^45^, can be released in EV-encapsulated forms and are capable of eliciting biological effects upon contact with sensitive cells^46^. We found that treatment with *Pkd1*-null cell EVs/exosomes increased the expression of TNF-α, IL-6, and MCP-1 in mouse IMCD cells and kidneys from *Pkd1*^*RC/RC*^ mice (Supplementary Fig. 14a–f), whereas treatment with GW4869 decreased the expression of TNF-α, IL-6, and MCP-1 in kidneys from *Pkd1*^*RC/RC*^ mice (Supplementary Fig. 13d–f). We further found that treatment with *Pkd1*-null cell EVs/exosomes increased macrophage populations in kidneys from *Pkd1*^*RC/RC*^ mice (Supplementary Fig. 15a) and treatment with GW4869 decreased macrophage populations in the pericystic region and interstitium in *Pkd1*^*RC/RC*^ kidneys and *Pkd1*^*flox/flox*^:*Pkhd1-Cre* kidneys as examined by F4/80 staining (Supplementary Fig. 15b, c). These results suggest that cystic EVs/exosome-encapsulated cytokines may stimulate the expression of cytokines and the recruitment of macrophages to promote cyst progression.

## Discussion

**Fig. 10:**
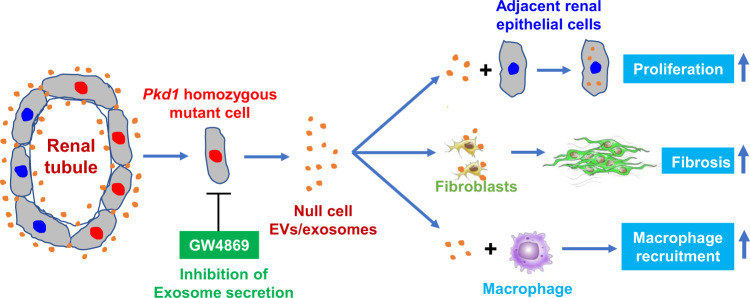
Working model of the “cystic extracellular vesicles/exosomes theory” in ADPKD. Renal cysts in ADPKD are proposed to be clonal in nature (derive from a single cell) and can arise from cells with inherited heterozygous germline mutations of *PKD1* or *PKD2* on one allele and a somatic mutation inactivating the remaining normal allele (two-hit model). After the second hit, the homozygous *PKD1* mutant renal epithelial cell may be via its secreted extracellular vesicles/exosomes, which can be secreted from apical and basolateral sides of cyst-lining epithelial cells, to affect the biology and function of neighboring cells, including heterozygous renal epithelial cells, fibroblasts, and macrophages during cyst initiation and expansion. In particular, cystic cell-derived EVs/exosomes could (1) lower the levels of polycystin to a critical threshold to promote *Pkd1* heterozygous renal epithelial cell proliferation and cyst growth (“threshold model”); (2) activate interstitial renal fibroblasts to promote renal fibrosis; and (3) induce the recruitment of macrophages to pericystic and interstitial regions in cystic kidneys. Inhibition of exosome biogenesis and release with GW4869 delays cyst growth in vivo. This “cystic extracellular vesicles/exosomes theory” addresses a long-time issue in PKD field of if and how ADPKD gene null renal epithelial cells affect the biology and function of neighboring cells, which integrates the “two-hit model” and “threshold model” together in renal cyst initiation and progression, and suggests a therapeutic potential for ADPKD treatment by targeting abnormal exosome secretion.

Over the past decade or so, EVs/exosomes have been extensively studied as a critical factor for pathophysiological processes of human diseases. However, the roles of EVs/exosomes in ADPKD and the in vivo mechanisms underlying the EVs/exosome-elicited action remain unknown. Most importantly, we do not know whether specific blockade of exosome production during disease has therapeutic effects. In this study, we provided evidence to support that cystic cell-derived EVs/exosomes are one of the key players to mediate the pathogenesis of ADPKD and proposed a “cystic EVs/exosomes theory” (Fig. 10). Particularly, we found that cystic cell-derived EVs/exosomes and urinary exosomes derived from ADPKD patients not only affected recipient cell function but also promoted cyst growth in *Pkd1* mutant mice and in 3D cultures by the downregulation of *Pkd1* gene expression and upregulation of miRNAs, including miR-200s and miR-21, leading to the activation of PKD-associated signaling pathways. In addition, treatment with cystic cell-derived EVs/exosomes induced: (1) the activation of fibroblasts and the expression of fibrotic markers to increase renal fibrosis; and (2) the expression of cytokines and the recruitment of macrophages to increase renal inflammation in cystic kidneys. We identified a positive feedback loop between miRNAs in cystic cells and miRNAs in EVs/exosomes as well as in recipient cells, representing one of the mechanisms for cystic cell-derived EVs/exosomes to promote cyst growth as well as renal inflammation and renal fibrosis in cystic kidneys. Furthermore, we found that the expression of genes associated with EVs/exosome biogenesis was upregulated in *Pkd1* mutant renal epithelial cells and tissues, suggesting that abnormal secretion of cystic renal epithelial cell EVs/exosomes may occur in cystic kidneys. Inhibition of exosome biogenesis/release with GW4869 delayed cyst growth in aggressive and milder ADPKD mouse models. This study addresses that *PKD* mutant renal epithelial cells could affect the cell function of neighboring cells that are not in direct contact with cells via secreted EVs/exosomes, and suggests a therapeutic potential for ADPKD treatment by targeting abnormal exosome secretion.

Renal cysts have been proposed to be derived from a single cell with heterozygous germline mutations and then a second hit to inactivate the remaining normal allele, resulting in clonal cyst expansion. A key question is after a second hit if the *Pkd1* homozygous mutant renal epithelial cell can affect the cell function of neighboring indirect-contact *Pkd1* heterozygous renal epithelial cells and fibroblasts, leading to cyst expansion. EVs/exosomes released from mammalian cells have been implicated as a tool for cell communication^47–49^. It has been reported that EVs/exosomes can be secreted from both apical and basolateral sites of epithelial cells^50^. We detected EVs/exosomes at both apical and basolateral sides on cyst-lining epithelial cells and normal renal tubular cells (Supplementary Fig. 16a). In addition, we did isolated EVs/exosomes from cyst fluids (Supplementary Fig. 16b). These results suggested that EVs/exosomes could be secreted not only from apical sides to cyst fluid but also from apical and basolateral sites of cystic renal epithelial cells to affect adjacent cell function during cyst progression. We found that cystic cell-derived EVs/exosomes are able to induce the activation of recipient cells, including *Pkd1* wild-type renal epithelial cells and fibroblasts, by downregulation of *Pkd1* expression (Fig. 2a, b), and activation of PKD-associated signaling pathways (Fig. 2c) and fibrotic markers (Fig. 5e–h), leading to cyst formation and renal fibrosis (Figs. 4,5). ADPKD urinary exosomes could also activate PKD signaling in recipient cells and promote cyst growth in 3D cultures (Fig. 3f, g). These results support that cystic cell-derived EVs/exosomes could affect the biology and function of recipient cells to further promote cyst progression.

Another question is how do cystic cell-derived EVs/exosomes activate PKD signaling in recipient cells? In ADPKD, in addition to the “two-hit hypothesis”, evidence supports a PKD “threshold model”, in that factors cause the amount or function of the PKD proteins to fall below a certain threshold, resulting in cyst formation^4^. Treatment with cystic cell-derived EVs/exosomes decreased *Pkd1* mRNA and proteins in recipient cells (Fig. 2a, b) and in cystic kidneys (Supplementary Fig. 7b), suggesting that EVs/exosomes may be a nongenetic factor that lowers the amount of polycystin 1 in the cystic kidneys. Once the level of PKD protein reaches a critical threshold, it may result in the activation of the known PKD-associated signaling pathways as shown in Figs. 2c and  4k, leading to increased renal epithelial cell proliferation and promote cyst growth (Figs. 3 and 4). These results suggested that the PKD “two-hit model” and “threshold model” are not mutually exclusive and may actually be affected with each other during the clonal expansion of cysts. In support of this, we found that treatment with cystic cell EVs/exosomes induced tubular dilation and small cyst formation in 3-month old *Pkd1*^*flox/+*^*:Pkhd1-Cre* mouse kidneys (Supplementary Fig. 8a). *Pkd1*^*flox/+*^*:Pkhd1-Cre* mice with *Pkd1* deletion in one allele developed normally with no renal cyst formation up to 1 year. Thus, renal cyst formation in this mouse model by 3-month old should be caused by the cystic cell EVs/exosome treatment that induced the downregulation of *Pkd1* (Supplementary Fig. 8c). However, treatment with cystic cell EVs/exosomes did not induce tubule dilation and cyst formation in 3-month-old wild-type mouse kidneys, suggesting that when both *Pkd1* wild-type alleles present this treatment did not result in the levels of *Pkd1* to reach the cystic threshold in vivo. Based on this result, a possible scenario in ADPKD kidneys is when a second hit occurs and results in a homozygous *PKD1-*null cell, its secreted EVs/exosomes may lower the levels of *PKD1* in the neighboring *PKD1* heterozygous renal cells and then both of these cells contribute to the focal cyst formation.

However, how cystic cell-derived EVs/exosomes regulate the expression of *Pkd1* in recipient cells is unknown. microRNAs function as sequence-specific inhibitors of gene expression^51^. Bioinformatics comparison of five different databases predicts that miR-200 family members directly bind to the two conserved binding sites in *Pkd1* 3’-UTR to inhibit its translation (Supplementary Fig. 9)^52^. In addition, miR-21 is upregulated in cystic kidneys and promotes cyst progression in ADPKD mouse models^53^. We found that both miR-200s and miR-21 were upregulated in cystic renal epithelial cells and tissues (Fig. 6). It has been highly recognized that the secreted EVs/exosomes should contain the miRNAs from the same cells^54^. The upregulation of miRNAs and miR21 in *Pkd1* mutant renal epithelial cells increased the amount of these miRNAs in cystic cell-derived EVs/exosomes (Fig. 6). Treatment with *Pkd1* mutant cell EVs/exosomes increased the expression of miR-200s and miR-21 in mouse IMCD cells (Supplementary Fig. 10), suggesting that a positive feedback loop may exist between cystic cell miRNAs and EVs/exosome miRNAs as well as neighboring cell miRNAs, which should be one of the mechanisms for cystic cell EVs/exosomes to induce the downregulation of *Pkd1* gene and the activation of PKD-associated pathways in recipient cells. However, in addition to miRNAs, EVs/exosomes can also deliver a diverse array of other biomolecules including messenger RNAs (mRNAs), proteins, and lipids^55^. Thus, future studies are needed to identify other mechanism(s) mediated by cystic cell-derived EVs/exosomes in recipient cells.

Renal cyst progression and expansion result in renal fibrosis in ADPKD. However, how clonal cyst formation and expansion affects the activation of interstitial fibroblasts is unknown. Sustained activation of fibroblasts which is a primary source of the scar-forming matrix proteins plays a key role in perpetuating renal fibrosis^56^. The activation of quiescent interstitial fibroblasts, to proliferating and excessively matrix-producing cells can be achieved through stimulation with growth factors and extracellular matrix signals, direct cell–cell contacts, and environmental conditions in renal disease^57^. We found that treatment with cystic cell-derived EVs/exosomes could induce fibroblast activation (Fig. 5), whereas inhibition of exosome biogenesis decreased renal fibrosis in cystic kidneys (Fig. 9e-h), suggesting a mechanism for renal fibrosis in ADPKD kidneys, in which cystic renal epithelial cells may regulate the activation renal fibroblasts via cystic cell secreted EVs/exosomes.

Macrophages are increased in pericystic and interstitial regions in cystic kidneys and promote cyst growth^44^. We found that treatment with cystic cell EVs/exosomes increased pericystic and interstitial macrophages in *Pkd1*^*RC/RC*^ kidneys compared to that in kidneys of the control mice (Supplementary Fig. 15a). In addition, we found that treatment with cystic cell exosomes increased the expression of TNF-α, IL-6, and MCP-1 in recipient cells and in cystic kidneys (Supplementary Fig. 14a–c). Both TNF-α and MCP-1 have been found to help the recruitment of macrophages to inflammatory sites and tissues^58^. Thus, cystic renal epithelial cell secreted EVs/exosomes may be via the upregulation of TNF-α and MCP-1 to increase the recruitment and/or the proliferation of macrophages in cystic kidneys. Cytokines, including TNF-α, IL-6 and MCP-1, can be released in EV-encapsulated forms and are capable of eliciting biological effects upon contact with sensitive cells^45,46^. Deciphering the regulatory mechanisms of EV encapsulation of cytokines should facilitate to identify the specific signal/factor from cystic cell EVs/exosomes that is involved in the induction of macrophage populations, leading to a better understanding of cell–cell communications in health and disease.

Polycystins are essential for normal blood vessel development and cardiovascular complications are an important cause of premature death and morbidity in ADPKD^59^. However, if and how the clonal nature of renal cystogenesis regulates blood vessel development and the cardiovascular complications in ADPKD is unknown. In addition to affect the adjacent cell biology and function, cystic cell EVs/exosomes may also enter the blood vessel. Interestingly, we found that treatment with cystic cell EVs/exosomes induced glomerular hypertrophy and promoted glomerular capillary loops to develop cyst-like structures in kidneys of *Pkd1*^*RC/RC*^ mice and *Pkd1*^*flox/+*^*:Pkhd1-Cre* mice (Supplementary Fig. 8e). This result suggests that circulating cystic cell exosomes might affect vascular endothelial cells, blood vessel development, and cardiovascular complications in ADPKD. Further testing of this hypothesis may shed significant insights into the mechanism of the vasculopathy in ADPKD.

Exosomes can be released from the cell by two mechanisms: constitutive or inducible. The constitutive secretion pathway is regulated by certain RAB GTPases (Rab27a/b, Rab11, and Rab35), heterotrimeric Gprotein, WNT5A, and glycosphingolipids^60–65^. On the other hand, the inducible secretion is regulated by stress stimuli, including aberrant intracellular calcium release, DNA damage, heat shock, hypoxia, and inflammatory stimulation^66^. We found that the expression of Rab27a and nSMase 2, the two key enzymes that regulate exosome secretion and release, was upregulated in postnatal *Pkd1* homozygous PN24 cells and cystic kidneys (Fig. 7a, b), suggesting that exosome biogenesis/release may be increased in *Pkd1* mutant renal epithelial cells and tissues. This result also suggests that the amounts of urinary exosomes produced by normal individuals and ADPKD patients should be different (Fig. 3a, b). However, due to the lack of an internal control to quantify this difference and the urinary contents, including exosomes, may be diluted if a person drinks more water, it may not be possible to use this character as a potential biomarker for disease progression in ADPKD. GW4869 is a commonly used pharmacological agent, which inhibits exosome biogenesis/release by blocking the ceramide-mediated inward budding of multivesicular bodies (MVBs) and the release of mature exosomes from MVBs^39,42^. We found that treatment with GW4869 significantly delayed cyst growth in *Pkd1*^*RC/RC*^ (Fig. 8a–g) and *Pkd1*^*flox/flox*^*:Pkhd1-Cre* mice (Supplementary Fig. 13a–e). These results suggested that the amelioration of renal cyst growth observed in GW4869-treated ADPKD mice should be ascribed to reduced generation of harmful exosomes from cystic cells. Treatment with cystic cell EVs/exosomes increased macrophages in cystic kidneys, whereas treatment with GW4869 decreased macrophage populations in kidneys from *Pkd1*^*RC/RC*^ mice and *Pkd1*^*flox/flox*^:*Pkhd1-Cre* mice (Supplementary Fig. 15b, c), supporting that cystic cell-derived exosomes contributed to the increase of macrophages in cystic kidneys. Systemic delivery of GW4869 at the doses used did not cause any cellular damage in the kidney and liver^67^. These results not only supported a direct role of abnormal exosome secretion in renal cyst formation but also suggested a beneficial effect of targeting exosome secretion in ADPKD treatment.

In conclusion, our study identified that EVs/exosomes are key regulators of cyst formation in ADPKD and support a “cystic EVs/exosomes theory”, in that EVs/exosomes derived from cystic renal epithelia cells could affect the biology of neighboring cells, including *Pkd1* heterozygous renal epithelial cells, fibroblasts, and microphages. Inhibition of exosome biogenesis/release with pharmacological agents, such as GW4869, could be a potential therapeutic strategy for ADPKD treatment.

## Methods

### Cell culture and reagents

Mouse IMCD3 cells were maintained at 37 °C in 5% CO_2_ in DMEM (Invitrogen) supplemented with 10% FBS. PH2 and PN24 cells (provided by S. Somlo through the George M. O’Brien Kidney Center, Yale University, New Haven, Connecticut, USA) were cultured at 33 °C in 5% CO_2_ in DMEM (Invitrogen) supplemented with 10% FBS and interferon-γ^68^. *Pkd1* WT and *Pkd1-* null MEK cells, derived from collecting ducts and sorted by the collecting duct marker dolichos biflorus agglutinin (DBA) from kidneys of WT and *Pkd1*-null mice, were maintained as previously described^10^. NRK-49F cells were maintained at 37 °C in 5% CO_2_ in DMEM (Invitrogen) supplemented with 10% FBS. GW4869 was purchased from SELLECK CHEMICALS and dissolved in DMSO (Sigma-Aldrich) at a stock solution of 10 mM. The stock solutions were stored at –20 °C.

The antibodies used for Western blot analysis included (a) anti-Rab27a (sc-74586), nSMase2 (sc-166637), anti-PCNA (sc25280), and anti-fibronectin (sc-59826), which were purchased from Santa Cruz Biotechnology Inc.; (b) anti-α-SMA (ab7817), anti-collagen 1 (ab34710), anti-TSG101 (ab125011), anti-ALIX (ab 275377), and CD63 (ab217345), which were purchased from abcam; (c) anti-STAT3 (no. 9139), anti-ERK (no. 4696), anti-S6 (no. 2217), anti-Rb (no. 9309), anti-AKT (no. 9272), anti-4EBP-1 (no. 9644), and the phosphorylated antibodies for STAT3-Y705 (no. 9131), ERK-T202/Y204 (no. 9101), S6-S235/236 (no. 2211), AKT-S473 (no. 9271), 4EBP-1-S65 (no. 9451), and Rb-S780 (no. 9307), which were purchased from Cell Signaling Technology; and (d) anti-actin antibody (A2228) and anti-tubulin (T7941) antibody, which were purchased from Sigma-Aldrich. In addition, the anti-PC1 antibody (7e12) was generated by Mayo Clinic^69^. The secondary antibodies, including donkey anti-rabbit IgG–horseradish peroxidase (sc-2313), donkey anti-goat IgG–horseradish peroxidase (sc-2020), and goat anti-mouse IgG–horseradish peroxidase (sc-2005), were purchased from Santa Cruz Biotechnology Inc.

### Western blot analysis

Cell pellets were collected and resuspended in lysis buffer (20 mM Tris-HCl, pH 7.4, 150 mM NaCl, 10% glycerol, 1% Triton X-100, 1 mM Na3VO4, 25 mM β-glycerolphosphate, 0.1 mM PMSF, Roche complete protease inhibitor set, and Sigma-Aldrich phosphatase inhibitor set). The resuspended cell pellet was vortexed for 20 s and then incubated on ice for 30 min and centrifuged at 20,000 × *g* for 30 min. The supernatants were collected for Western blot analysis.

### Histology and immunohistochemistry

Paraffin-embedded sections (4 μm) were subjected to H&E staining and immunohistochemistry. For staining, a monoclonal mouse anti-nSMase2 antibody (1:50 dilution) and Rab27a (1:50 dilution), biotinylated secondary antibody (1:100 dilution), and DAB substrate system were used. Kidney sections were counterstained by hematoxylin. Images were analyzed with a Nikon Eclipse 80i microscope. Tissue fibrosis was assessed using Picrosirius Red (Abcam, ab150681) as per the manufacturer’s instruction.

### Immunofluorescence staining

For Ki67 and collagen 1 staining, rabbit anti-Ki67, rabbit anti- collagen 1 antibody, and Alexa Fluor 488 anti-rabbit IgG secondary antibody were used. For PCNA, α-SMA, fibronectin staining, mouse anti-PCNA, mouse anti-α-SMA antibody, mouse anti-fibronectin, and Alexa Fluor 555 anti-mouse IgG secondary antibody were used. For Rab27a and nSMase 2 staining, mouse anti-Rab27a antibody, mouse anti-nSMase 2, as wel as LTL and DBA were used. Macrophages were detected by immunofluorescence staining (IF staining) with a pan-macrophage marker, F4/80. After antigen retrieval, tissue sections were incubated with a rat anti-mouse F4/80 antibody (14-4801-82; eBioscience Inc.; 1:100 dilution) overnight, and then were incubated with Fluro-555 anti-rat IgG secondary antibody and mounted in Prolong Gold Antifade reagent with DAPI (Invitrogen). Images were analyzed using a Nikon Eclipse 80i microscope.

### Urine exosome isolation

Approximately 100 ml of first-void urines were collected from 5 healthy human volunteers and 5 ADPKD patients between the ages of 18 and 40 years with an estimated glomerular filtration rate (eGFR) between 40 and 80 mL/min/1.73 m^2^, evaluated by the abbreviated Modification of Diet in Renal Disease (MDRD) formula. Urine was centrifuged at 17,000 × *g* for 15 min at 4 °C to remove urinary sediments, including whole cells, large membrane fragments, and other debris. An aliquot of the supernatant was removed and the remaining supernatant was centrifuged at 200,000 × *g* for 1 h at 4 °C to obtain low-density membranes. The 200,000 × *g* supernatants were then removed and replaced with an additional 17,000 × *g* supernatant and ultracentrifuged again. This centrifugation step was repeated 3–5 more times to harvest the low-density exosome pellets from 96 ml of 17,000 × *g* supernatant. Pellets were resuspended in 200: 1 of isolation solution (10 mM triethanolamine/250 mM sucrose (pH 7.6); 0.5 mM PMSF,and 1 μm Leupeptin), and pooled. After removing 10:1 for bicinchoninic acid (BCA) protein assay (Pierce), the remaining suspension was added to an equal volume of 2 × Laemmli sample buffer containing 60 mg/ml DTT and heated at 60 °C for 10 min. These samples were divided into aliquots and stored at −80 °C until use.

### 3D culture and cyst measurement

The type-I rat-tail collagen (1.5 mg/ml, BD Biosciences, collagen I) solutions with desired concentration in DMEM-F12 containing 100 U/ml penicillin, 100 U/ml streptomycin, 10 mM Hepes (pH 7.2), and 1.2 mg/ml NaHCO3 were prepared on ice, and then mixed with mouse IMCD cells plus EVs/exosomes (100 ug/ml) to make a cell–collagen suspension at a density of 1 × 10^4^ cells/ml. Aliquots of 0.4 ml of the cell–collagen suspension were plated in a 24-well plate and incubated for 30 min in a 37 °C, 5% CO_2_ incubator to allow collagen solidified and then overlaid with 1.5 ml of medium plus EVs/exosomes (100 ug/ml), which was replaced every other day. The plates were kept in a tissue culture incubator (37 °C, 5% CO_2_, and 100% humidity). Cysts were allowed to grow for 8 days in culture and cystic structures were assessed and photographed by light microscopy.

The mixing of EVs/exosomes with mouse IMCD cells at day 0 should facilitate the entry of EVs/exosomes to the recipient cells at this stage. However, whether EVs/exosomes added every other day to the collagen gel could penetrate the gel to reach the embedded cells to fuse with and deliver their content to the latter is uncertain. If not, the mixing of EVs/exosomes with IMCD cells at day 0 should be necessary to promote cyst formation in 3D cultures.

### EV/exosome purification, analysis, and labeling

Serum-free conditioned medium was collected after cells were cultured for 48 h, and EVs/exosomes were purified by several centrifugation and filtration steps. Briefly, the conditioned media were centrifuged at 300 × *g* for 10 min, 2000 *×* *g* for 10 min, and 10,000 × *g* for 40 min. Then, the supernatant was ultracentrifuged at 100,000 g for 70 min, followed by an additional washing step of the EV/exosome pellets with PBS at 100,000 × *g* for 70 min (Ultracentrifuge, Thermo Scientific). The protein content was measured using MicroBCA protein assay (Thermo Scientific). Cell-derived EVs/exosomes were analyzed for the presence of the exosomal marker protein CD63 (ab217345; Abcam) by Western blot. For EV/exosome-uptake experiments, PN24 cell-derived EVs/exosomes were labeled with the PKH67 Green Fluorescent Cell Linker Kit (Sigma-Aldrich) according to the manufacturer’s protocol with minor modifications. EVs/exosomes diluted in PBS were added to 1 ml of Diluent C (Sigma-Aldrich). In parallel, 4 μl of PKH67 dye was added to 1 ml of Diluent C and incubated with the EV/exosome solution for 4 min. To bind excess dye, 2 ml of 0.5% BSA/PBS was added. The labeled EVs/exosomes were washed at 100,000 g for 1 h, and the EV/exosome pellets were diluted in 100 μl of PBS and used for uptake experiments and biodistribution analysis.

To assess the uptake of EVs/exosomes to recipient cells, IMCD cells were seeded onto coverslips in a 6-well plate and allowed to attach overnight. The next day, cells were washed and then cultured in fresh media plus PN24 cell-derived EVs/exosomes (100 μg/ml) at 37 °C. After 24 h, cells were washed with PBS three times, fixed in 4% paraformaldehyde for 10 m at room temperature, and washed with PBS again. Cells were labeled with ZO-1 monoclonal antibody (ZO1-1A12) (Product # 33-9100), followed by donkey anti-mouse IgG (H+L) secondary antibody, Alexa Fluor Plus 555. Confocal microscopy was performed to visualize the localization of PKH67-labeled EVs/exosomes in IMCD cells (LSM980 Confocal Microscope, ZEISS, White Plains, NY, United States). Z-stack confocal imaging was performed on serial slices of IMCD cells and image processing was accomplished using Imaris software (v.8.2.1, Bitplane).

### Characterization of purified EVs/exosomes

EV/exosome samples were placed on 100-mesh carbon-coated, formvar-coated copper grids treated with poly-l-lysine for ~30 min. Samples were then negatively stained with Millipore-filtered aqueous 1% uranyl acetate for 1 min. Stain was blotted dry from the grids with filter paper and samples were allowed to dry. Samples were then examined in transmission electron microscope at an accelerating voltage of 80 Kv. For immunogold labeling, purified EVs/exosomes suspended in PBS were placed on formvar carbon-coated nickel grids, blocked, and incubated with antibody CD63, followed by incubation with the anti-Rabbit secondary antibody conjugated with protein A-gold particles (10 nm). Each staining step was followed by five PBS washes and ten ddH_2_O washes before contrast staining with 2% uranyl acetate. The size and concentration of EVs/exosomes purified from cell culture supernatants were determined using a NanoSight NS300 (Malvern Instruments), which is equipped with fast video capture and particle-tracking software.

### MTT assays

Cell proliferation was measured using an MTT-based kit (Sigma-Aldrich), according to the manufacturer’s instructions.

### Quantitative reverse-transcription polymerase chain reaction (qRT-PCR)

Total RNA was extracted using the RNeasy Plus Mini Kit (Qiagen) or Trizol reagents. Total RNA (1 μg) was used for RT reactions in a 20-μl reaction to synthesize cDNA using an iScript cDNA Synthesis Kit (Bio-Rad) or MicroRNA first-strand synthesis kit (Takara Bio). RNA or miRNA expression profiles were analyzed by real-time PCR using iTaq SYBR Green Supermix with ROX (Bio-Rad) in an iCycler iQ Real-Time PCR Detection System. The complete reactions were subjected to the following program of thermal cycling: 40 cycles of 10 s at 95 °C and 20 s at 60 °C. A melting curve was run after the PCR cycles, followed by a cooling step. Each sample was run in triplicate in each experiment, and each experiment was repeated 3 times. Expression levels of target genes were normalized to the expression level of actin. Expression levels of target miRNA were normalized to the expression level of U6. All primers used are listed in Supplementary Table 1.

### Mouse strains and treatments

One-month-old 129 S/6 *Pkd1*^*RC/RC*^ mice were treated with cystic cell EVs/exosomes (200 ug in sterile PBS), wild-type cell EVs/exosomes (200 ug in sterile PBS), and PBS (control) by intravenous injection three times per week up to 3 months and kidneys were harvested from these mice 1 day after the last treatment. *Pkd1*^*RC/RC*^ mice were also treated with GW4869 (5 ug/g) or DMSO by daily intraperitoneal injection from 1 to 3 months. The kidneys were harvested at 3 month for further analysis. *Pkd1*^*flox/flox*^*:Pkhd1-Cre* mice were generated by cross-breeding *Pkd1*^*flox/+*^ *Pkhd1-Cre* female mice with *Pkd1*^*flox/+*^*: Pkhd1-Cre* male mice. Each neonate was intraperitoneally injected daily with GW4869 (5 ug/g) or DMSO from postnatal day 7 (P7) to P21. The kidneys were harvested and analyzed at PN25. Littermate controls were used in all animal experiments. Wild-type mice were treated with cystic cell EVs/exosomes (200 ug in sterile PBS) or PBS by intravenous injection three times per week from PN7 to 3-month old. The kidneys were harvested from 3-month-old mice for further analysis. All experiments involving animals were conducted under the approval of Mayo Clinic IACUC.

### Biodistribution of injected exosomes

To assess the biodistribution of cystic cell-derived EVs/exosomes, 6–8-week-old male *Pkd1*^*RC/RC*^ mice were treated with 200 μg of PKH67-labeled exosomes by intravenous injection in a volume of 200 μl of PBS and the mouse was also injected with 200 μL of PBS as background control. The *Pkd1*^*RC/RC*^ mice were sacrificed after 24 h post-EVs/exosome administration then the kidneys were harvested and analyzed by using in vivo imaging system (IVIS PerklinElmer, MA, USA).

### Measurement of cyst area

The cyst volume was quantified in whole kidney after H&E staining using Image-Pro Plus v5 software (Media Cybernetics). The cyst area was calculated as (cyst area/total area) × 100%. Three sections from both kidneys were analyzed for each mouse.

### Quantitative blood urea nitrogen (BUN) determination

Serum samples were first diluted 4-fold in distilled water prior to assay. Next, 5 μl of water (blank), 5 μl of standard (50 mg/dl), and 5 μl of samples were transferred in triplicate into wells of a clear-bottom 96-well plate. About 200 μl of working reagent was added and tapped lightly to mix, and the samples were incubated for 20 min at room temperature. Optical density was read at 520 nm.

### Ultra-high-Field MRI for mice

All imaging studies were performed on live animals under isoflurane anesthesia, with a Bruker AVANCEIII-700 (16.4 T) vertical-bore two-channel multinuclear spectrometer. Axial and coronal images were acquired with respiratory gating and spin-echo multi-slice sequence. Total kidney volumes were calculated from MR images using the Analyze 12.0 software.

### Statistics and reproducibility

All data presented were repeated at least three times independently with similar results. All statistical data are presented as mean ± SEM. All statistical analyses were performed using SPSS Statistics 22 software. *P* values were calculated by 2-tailed unpaired Student’s *t-*test and 1-way ANOVA; and a P value less than 0.05 was considered significant.

### Study approval

All animal protocols were approved by and conducted in accordance with Laboratory Animal Resources of Mayo Clinic and Institutional Animal Care and Use Committee regulations.

Urine specimens were obtained from the biobank of the Mayo Clinic PKD Repository, which is approved by the institutional review board (IRB), in accordance with FDA code 21 CFR 56. ADPKD patients and normal volunteers provided informed consent prior to the collection of urine and medical information. Urine samples and data were de identified.

### Reporting summary

Further information on research design is available in the Nature Research Reporting Summary linked to this article.

## Supplementary information

Supplementary Information

Reporting Summary

## Data Availability

All relevant data are available from the authors. A source data file has been included that contains all raw data underlying all reported averages in graphs and charts, as well as all uncropped versions of any gels or blots presented in the figures. Source data are provided with this paper.

## Source data

Source Data
