# Supplementary material for: Extracellular vesicles and exosomes generated from cystic renal epithelial cells promote cyst growth in autosomal dominant polycystic kidney disease
Source: Nat Commun. 2021 Jul 27;12:4548. doi: 10.1038/s41467-021-24799-x (PMC8316472; doi:10.1038/s41467-021-24799-x)
Supplement: Supplementary file 1 — Supplementary Information [file 41467_2021_24799_MOESM1_ESM.pdf]

Supplementary figures and figure legends

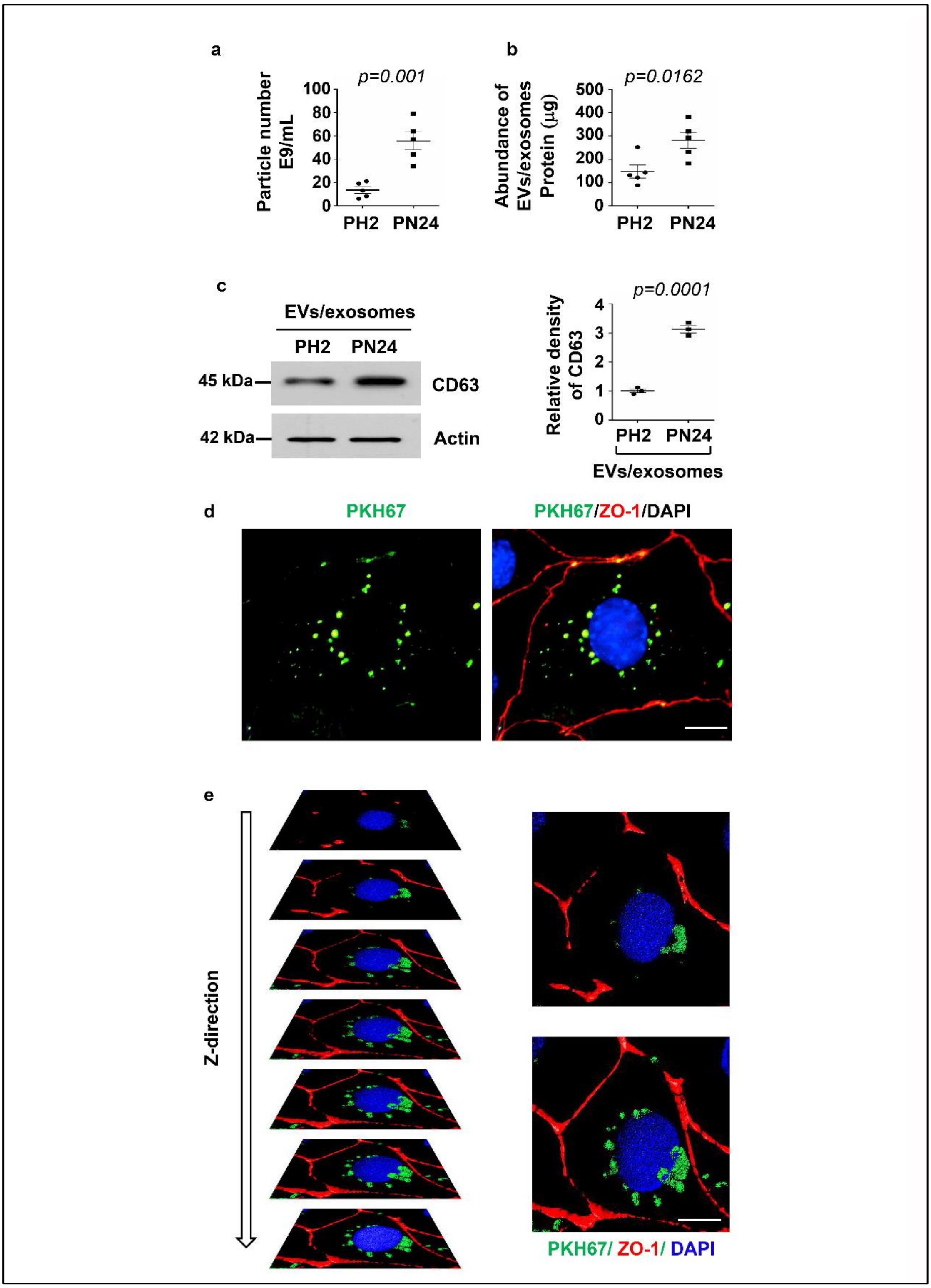

Supplementary Figure 1. The EVs/Exosome particles derived from PN24 cells and the levels of CD63 in those particles were increased.

(**a**) The EVs/Exosomes particles derived from PN24 cells were increased compared to those derived from PH2 cells as examined by Nanoparticle analysis. Data were analyzed from five experiments.

(b) The protein contents of the EVs/exosomes derived from PH2 and PN24 cells were examined by using BCA protein assay kit. Data were analyzed from five experiments.

(**c**) The levels of CD63 in PN24 cell derived EVs/Exosomes were compared to those in PH2 cell derived EVs/Exosomes by Western blot analysis, in which all of the analyses in **a** to **c** were adjusted with the same cell numbers of both cells used to collect EVs/Exosomes. Data were analyzed from three experiments.

(**d**) Representative images of mouse IMCD cells treated with PKH67-labeled EV/exosomes (green) and stained with ZO-1, a surface maker. Scale bars, 10 μm.

(**e**) Representative confocal images of mouse IMCD cells treated with PKH67-labeled EV/exosomes (green) and stained with ZO-1. Z-stack showing the distribution of PKH67-labeled EV/exosomes (green) within mouse IMCD cells bordered by ZO-1. Scale bars, 10 μm.

All statistical data are represented as mean ± SEM, and p-values are calculated by unpaired Student's t-test.

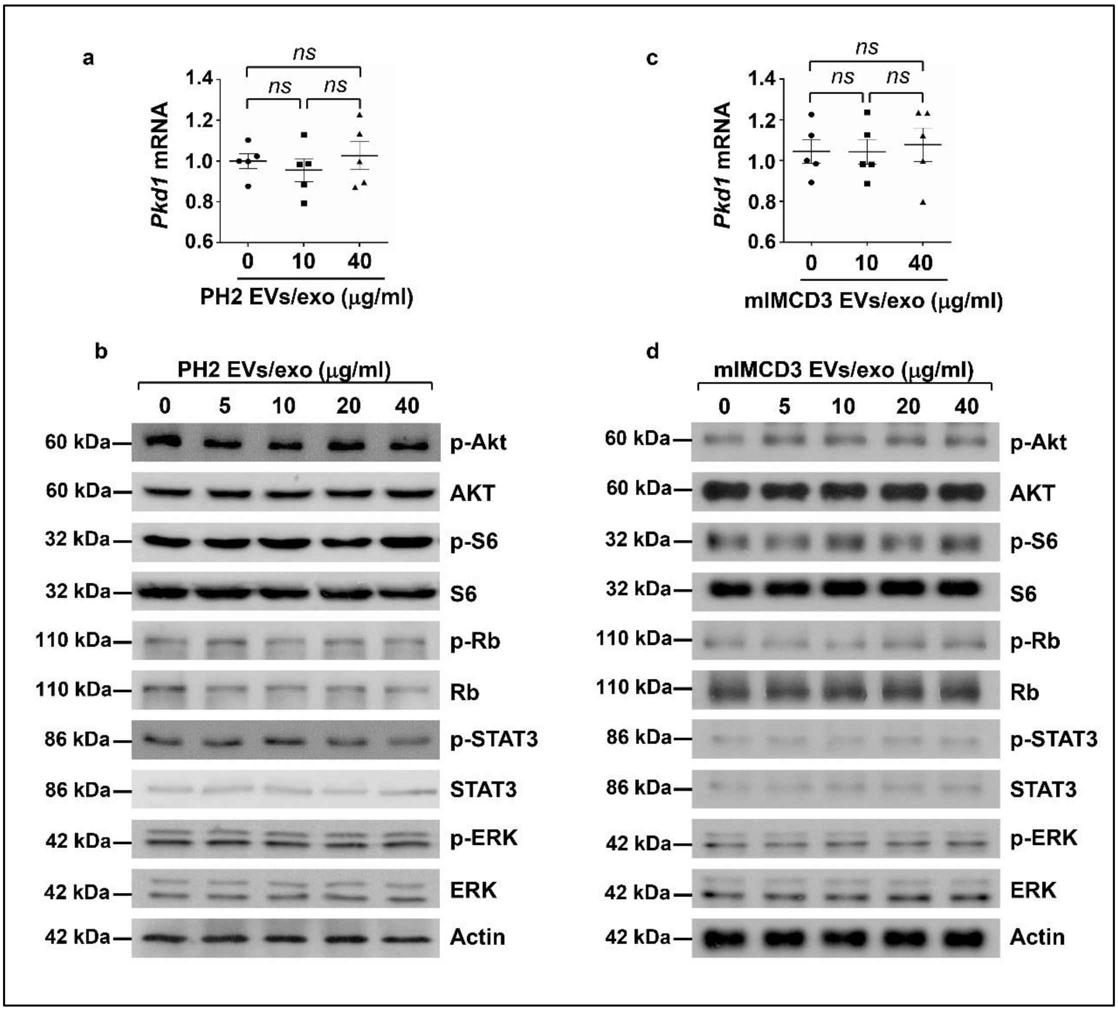

Supplementary Figure 2. Treatment with PH2 cell derived EVs/exosomes did not affect the expression of *Pkd1* gene and induce the activation of PKD-associated signaling pathways in mouse IMCD3 cells.

(**a**) qRT-PCR analysis of *Pkd1* mRNA expression in mIMCD3 cells treated with PH2 cell-derived EVs/exosomes.

(**b**) Western blot analysis of the phosphorylation and total proteins of AKT, mTOR, S6, Rb, STAT3 and ERK in mIMCD3 cells treated with PH2 cell-derived EVs/exosomes.

(**c**) qRT-PCR analysis of *Pkd1* mRNA expression in mIMCD3 cells treated with mIMCD3 cell-derived EVs/exosomes.

(**d**) Western blot analysis of the phosphorylation and total proteins of AKT, mTOR, S6, Rb, STAT3 and ERK in mIMCD3 cells treated with mIMCD3 cell-derived EVs/exosomes. n = 3 independent experiments in ***a*** and ***c***. All statistical data are represented as mean ± SEM, and p-values are calculated by one-way ANOVA followed by Tukey's post hoc test.

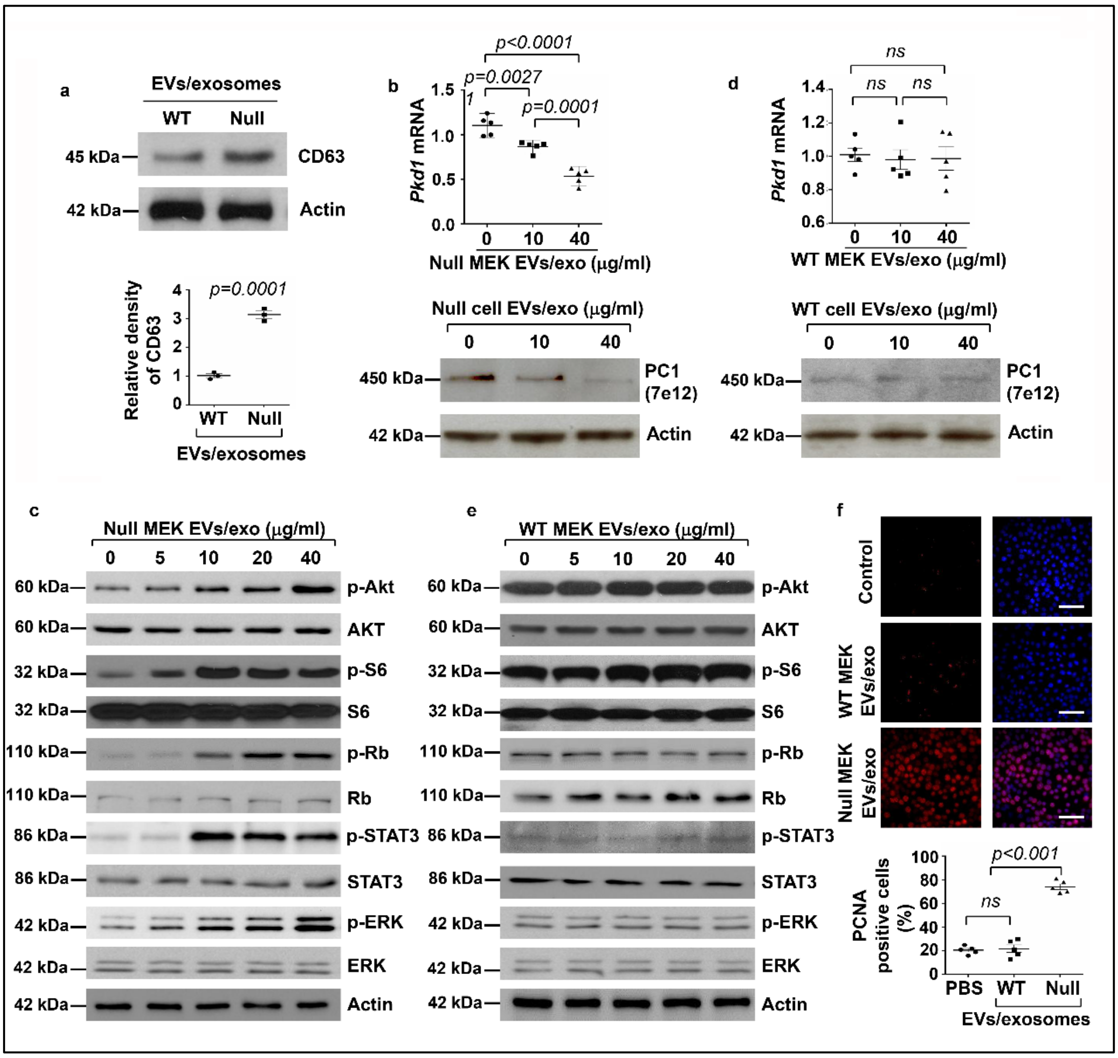
a
EVs/exosomes
WT
Null
45 kDa
CD63
42 kDa
Actin
Relative density of CD63
p=0.0001
WT
Null
EVs/exosomes
b
p<0.0001
p=0.0027
p=0.0001
Pkd1 mRNA
0
10
40
Null MEK EVs/exo (μg/ml)
Null cell EVs/exo (μg/ml)
450 kDa
PC1 (7e12)
42 kDa
Actin
d
ns
ns
ns
Pkd1 mRNA
0
10
40
WT MEK EVs/exo (μg/ml)
WT cell EVs/exo (μg/ml)
450 kDa
PC1 (7e12)
42 kDa
Actin
c
Null MEK EVs/exo (μg/ml)
0
5
10
20
40
60 kDa
p-Akt
60 kDa
AKT
32 kDa
p-S6
32 kDa
S6
110 kDa
p-Rb
110 kDa
Rb
86 kDa
p-STAT3
86 kDa
STAT3
42 kDa
p-ERK
42 kDa
ERK
42 kDa
Actin
e
WT MEK EVs/exo (μg/ml)
0
5
10
20
40
60 kDa
p-Akt
60 kDa
AKT
32 kDa
p-S6
32 kDa
S6
110 kDa
p-Rb
110 kDa
Rb
86 kDa
p-STAT3
86 kDa
STAT3
42 kDa
p-ERK
42 kDa
ERK
42 kDa
Actin
f
Control
WT MEK EVs/exo
Null MEK EVs/exo
PCNA positive cells (%)
p<0.001
ns
PBS
WT
Null
EVs/exosomes

Supplementary Figure 3. Treatment with *Pkd1*-null MEK cell derived EVs/exosomes decreased the expression of *Pkd1* gene and induced the activation of PKD-associated signaling pathways in mouse IMCD3 cells.

(**a**) Western blot analysis of the levels of CD63 in *Pkd1* null MEK cell derived EVs/Exosomes compared to those in *Pkd1* wild type MEK cell derived EVs/Exosomes. This analysis was adjusted with the same cell numbers of both cell types used to collect EVs/Exosomes. Data were analyzed from three experiments.

(**b**) qRT-PCR and Western blot analysis of *Pkd1* mRNA and protein in mIMCD3 cells treated with *Pkd1*-null MEK cell-derived EVs/exosomes. Data were analyzed from five experiments.

(**c**) Western blot analysis of the phosphorylation and total proteins of AKT, mTOR, S6, Rb, STAT3 and ERK in mIMCD3 cells treated with *Pkd1*-null MEK cell-derived EVs/exosomes.

(**d**) qRT-PCR analysis and Western blot of *Pkd1* mRNA and protein in mIMCD3 cells treated with *Pkd1*-wild type MEK cell-derived EVs/exosomes. Data were analyzed from five experiments.

(**e**) Western blot analysis of the phosphorylation and total proteins of AKT, mTOR, S6, Rb, STAT3 and ERK in mIMCD3 cells treated with *Pkd1*-wild type MEK cell-derived EVs/exosomes.

(**f**) Immunostaining for PCNA in mIMCD3 cells treated *Pkd1* wild type and null MEK cell derived EVs/exosomes (40 μg/ml) for 48 h. Cells were co-stained with DAPI to visualize the nuclei. Data were analyzed from five experiments. Scale bars, 100 μm.

All statistical data are represented as mean ± SEM in **a**, **b**, **d** and **f**, and p-values are calculated by one-way ANOVA followed by Tukey's post hoc test in b, d, f, and by two-tailed unpaired t tests in **a**.

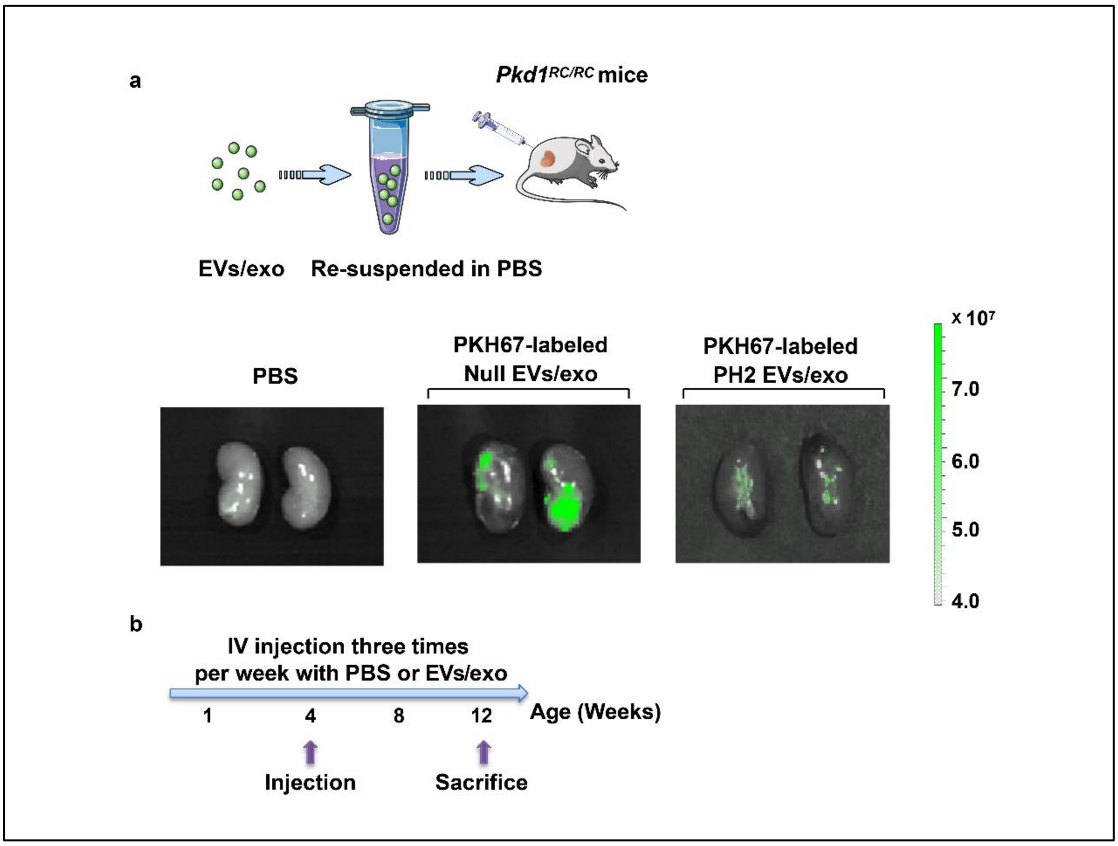

Supplementary Figure 4. Treatment with *Pkd1*-null cell derived exosomes *in vivo*. (**a**) EVs/exosomes (200 μg) were re-suspended in sterile PBS labeled with PKH67 and were intravenously (IV) injected (200 μg of purified EVs/exosomes from PN24 and PH2 cells) into *Pkd1*$^{RC/RC}$ mice. Kidneys were harvested after 24 hours injection for *ex vivo* imaging. The fluorescent signal of PKH67-labeled PN24 (*middle panel*) and PH2 (*right panel*) cell derived exosomes was detected with the *in vivo* imaging system (IVIS).
(**b**) Schematic diagram of experimental design. *Pkd1*$^{RC/RC}$ mouse were intravenous injected with PBS or exosomes (200 ug) three times per week from 4 weeks to 12 weeks, and 3 months old kidneys were harvested and analyzed.

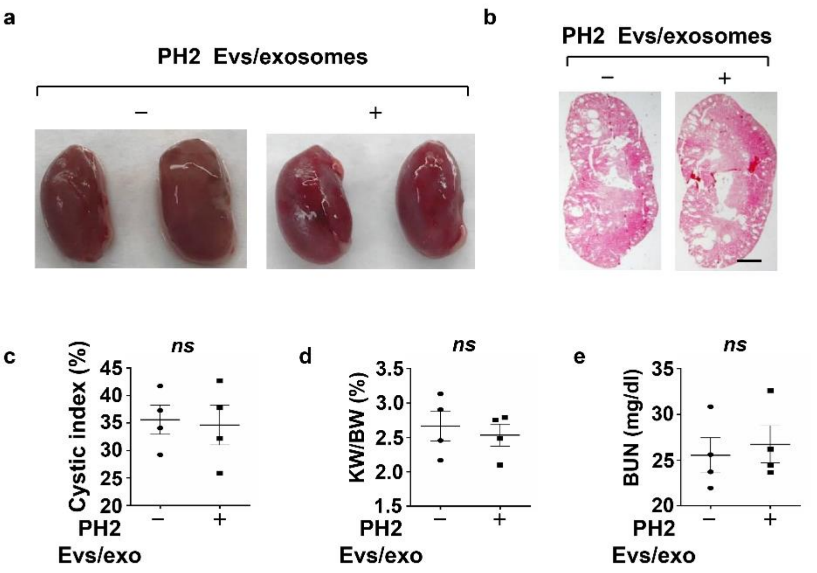

Supplementary Figure 5. Treatment with PH2 cell derived EVs/exosomes did not promote cyst growth *in vivo*.

(**a**) Images of kidneys from *Pkd1*$^{RC/RC}$ mice treated with or without EVs/exosomes from PH2 cells.

(**b**) Histological examination of kidneys from *Pkd1*$^{RC/RC}$ mice treated with or without EVs/exosomes from PH2 cells. Scale bars, 1 mm.

(**c**) Cyst index of kidneys from *Pkd1*$^{RC/RC}$ mice treated with WT EVs/exosomes (n = 4) or PBS (n = 4).

(**d** and **e**) Treatment with PH2 EVs/exosomes did not result in the increase of KW/BW ratios (**d**) and BUN levels (**e**) in *Pkd1*$^{RC/RC}$ mice compared to those in kidneys from control mice treated with PBS.

All statistical data are represented as mean ± SEM, and p-values are calculated by unpaired Student’s t-test.

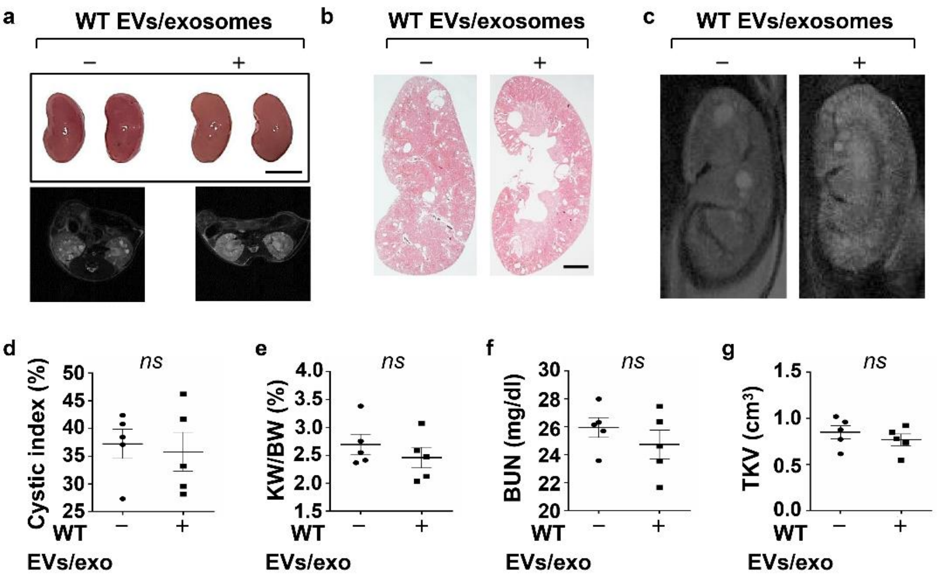

Supplementary Figure 6. Treatment with WT cell derived EVs/exosomes did not promote cyst growth *in vivo*.

(**a**) Images of kidneys (top panel) and axial MRI images (bottom panel) from *Pkd1*$^{RC/RC}$ mice treated with or without EVs/exosomes from mouse IMCD3 cells. Scale bars, 5 mm.

(**b**) Histological examination of kidneys from *Pkd1*$^{RC/RC}$ mice treated with or without EVs/exosomes from mIMCD3 cells. Scale bars, 1 mm.

(**c**) MRI images from *Pkd1*$^{RC/RC}$ mice treated with or without EVs/exosomes from mIMCD3 cells.

(**d**) Cyst index of kidneys from *Pkd1*$^{RC/RC}$ mice treated with WT EVs/exosomes (n = 5) or PBS (n = 5).

(**e** and **f**) Treatment with WT EVs/exosomes did not result in the increase of KW/BW ratios (**e**) and BUN levels (**f**) in *Pkd1*$^{RC/RC}$ mice compared to those in kidneys from control mice treated with PBS.

(**g**) Total kidney volume was calculated by MRI scan in each group.

All statistical data are represented as mean ± SEM, and p-values are calculated by unpaired Student’s t-test.

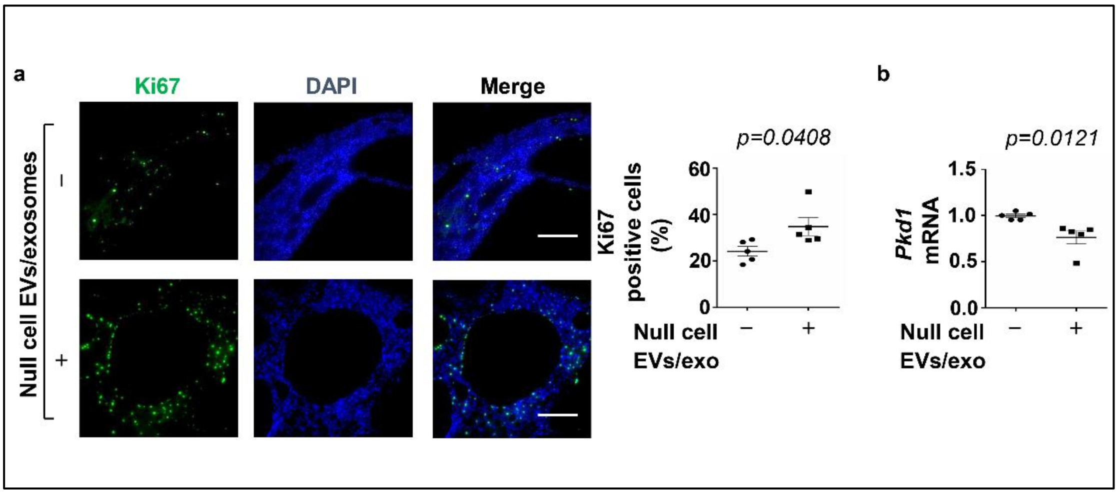

Supplementary Figure 7. Treatment with *Pkd1*-null cell derived EVs/exosomes promoted renal cell proliferation *in vivo*.

(**a**) Treatment with *Pkd1*-null EVs/exosomes increased cyst-lining and interstitial cell proliferation in kidneys from *Pkd1*$^{RC/RC}$ mice as detected with Ki67 staining. Scale bars, 100 µm. The percentage of Ki67 positive cells was calculated from an average of 1,000 nuclei per mouse kidney section.

(**b**) qRT-PCR analysis of the expression of *Pkd1* mRNA in kidneys from *Pkd1*$^{RC/RC}$ mice treated with *Pkd1*-null cell EVs/exosomes or PBS.

n = 5 independent experiments. All statistical data are represented as mean ± SEM, and p-values are calculated by unpaired Student's t-test.

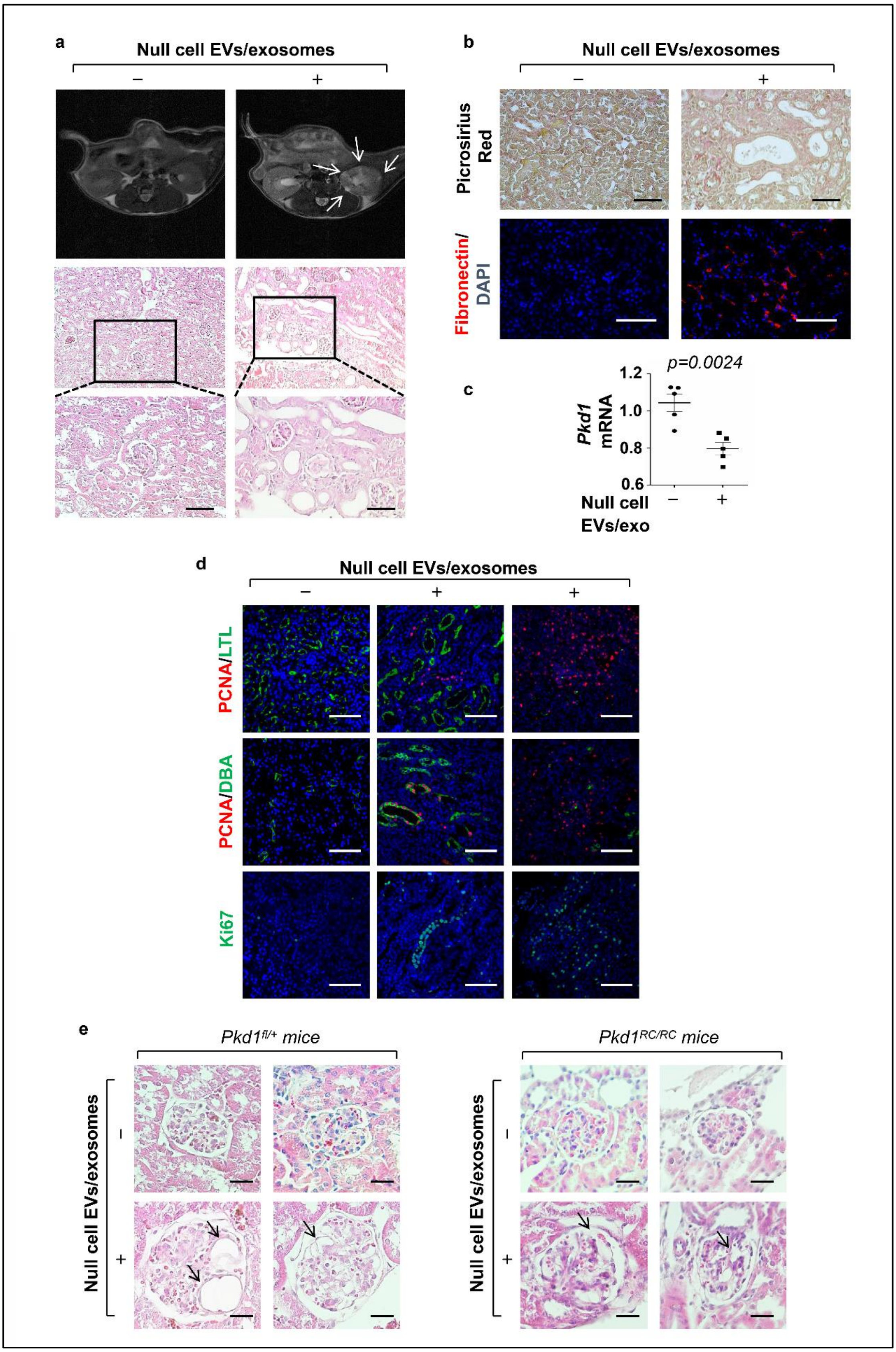

a
Null cell EVs/exosomes
–
+
b
Null cell EVs/exosomes
–
+
Picrosirius Red
Fibronectin/DAPI
c
p=0.0024
Pkd1 mRNA
1.2
1.0
0.8
0.6
Null cell EVs/exo
–
+
d
Null cell EVs/exosomes
–
+
+
PCNA/LTL
PCNA/DBA
Ki67
e
Pkd1fl/+ mice
Pkd1RC/RC mice
Null cell EVs/exosomes
–
+

Supplementary Figure 8. Treatment with *Pkd1*-null cell derived EVs/exosomes promoted cyst growth in *Pkd1*$^{flox/+}$:*Pkhd1-Cre* kidneys.

(**a**) MRI images of each group (top panel). Treatment with *Pkd1*-null cells EVs/exosomes induced tubular dilation and small cyst formation (arrows). Histological examination of cyst formation in kidneys from *Pkd1*$^{flox/+}$:*Pkhd1-Cre* mice treated with or without cystic cell EVs/exosomes (bottom panel). Scale bars, 50 μm.

(**b**) Picrosirius red staining of kidneys from *Pkd1*$^{flox/+:}$*Pkhd1-Cre* mice treated with or without *Pkd1*-null cell EVs/exosomes (top panel). Scale bars, 50 μm. Fibronectin immunofluorescent staining of kidneys from *Pkd1*$^{flox/+}$:*Pkhd1-Cre* mice treated with or without *Pkd1*-null cell EVs/exosomes (bottom panel). Scale bars: 100 μm.

(**c**) Treatment with *Pkd1*-null cell derived EVs/exosomes decreased the expression of *Pkd1* mRNA in *Pkd1*$^{flox/+}$:*Pkhd1-Cre* kidneys compared to that in kidneys from control mice treated with PBS (n=5).

(**d**) Immunostaining for PCNA and Ki67 as well as DBA and LTL staining of kidneys from *Pkd1*$^{flox/+}$:*Pkhd1-Cre* mice treated with or without *Pkd1*-null cell EVs/exosomes. Scale bars, 100 μm.

(**e**) Enlarged glomeruli and cyst-like structures in glomerular capillary loops were found in kidneys from *Pkd1*$^{flox/+}$:*Pkhd1-Cre* mice and *Pkd1*$^{RC/RC}$ mice treated with *Pkd1*-null cell EVs/exosomes. Scale bars, 25 μm.

All statistical data are represented as mean ± SEM, and p-values are calculated by unpaired Student’s t-test.

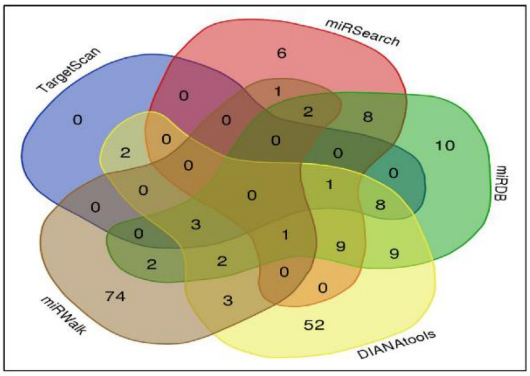

Names	Elements
DIANAtools	
TargetScan	mmu-miR-200b-3p
miRDB	mmu-miR-200c-3p
miRWalk	mmu-miR-429-3p

Supplementary Figure 9. Bioinformatic comparison of five different databases predicts that miR-200 family members directly bind to *Pkd1* 3'-UTR and inhibit its translation.

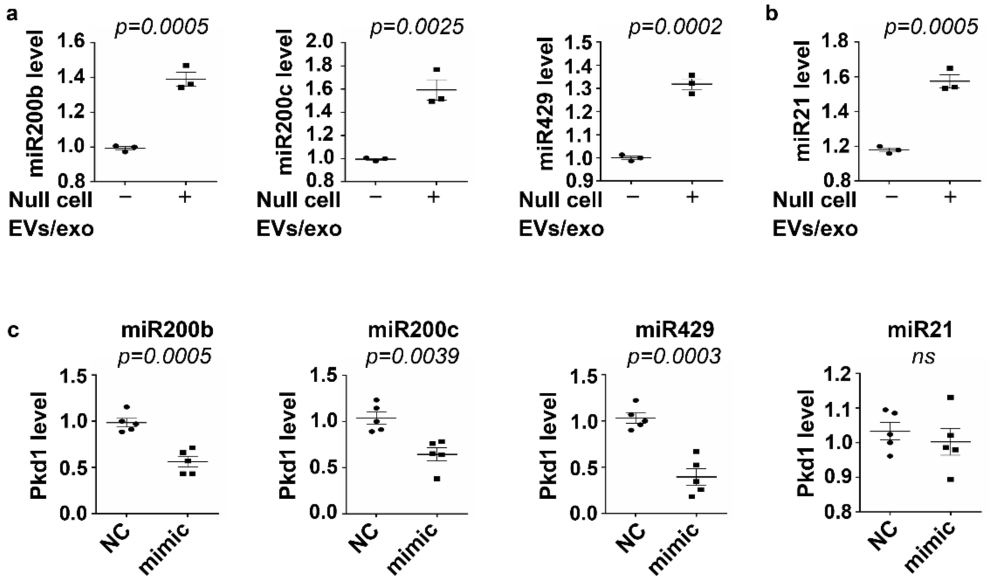

Supplementary Figure 10. Treatment with cystic cell EVs/exosomes increased the expression of miRNAs in mouse mIMCD3 cells.

(**a** and **b**) qRT-PCR analysis of the expression of miR200b, miR200c and miR429 miRNA (**a**) and miR21 (**b**) in mIMCD3 cells treated with or without cystic cell EVs/exosomes. Data were analyzed from three experiments.

(c) qRT-PCR analysis of *Pkd1* mRNA levels of in mIMCD3 cells transfected with miR-200 family mimics and miR21 mimic or negative control (N.C.). Data were analyzed from five experiments.

All statistical data are represented as mean ± SEM, and p-values are calculated by unpaired Student's t-test.

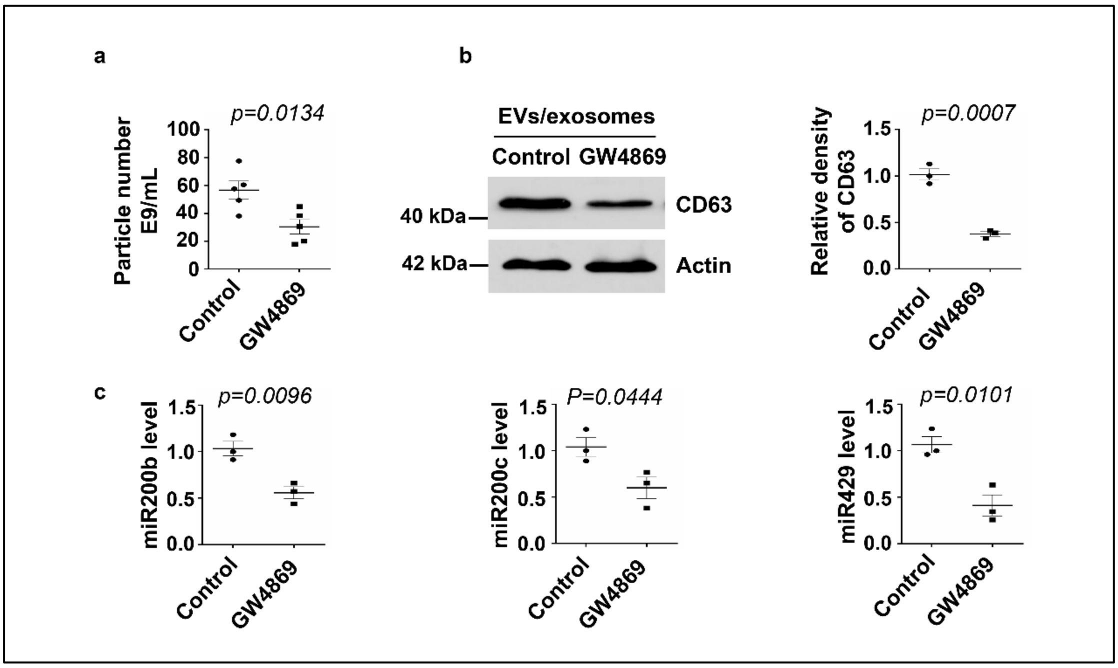

Supplementary Figure 11. Treatment with GW4869 decreased EVs/Exosome particles derived from PN24 cells and the levels of CD63 and miRNAs in those particles.

(**a**) Treatment with GW4869 decreased the EVs/Exosomes particles derived from PN24 cells compared to those derived from untreated control cells as examined by Nanoparticle analysis. Data were analyzed from five experiments

(b) Treatment with GW4869 decreased the levels of CD63 in PN24 cell derived EVs/Exosomes compared to those derived from untreated control cells as examined by Western blot analysis. The analyses in **A** and **B** were adjusted with the same cell numbers of both cells used to isolate EVs/Exosomes. Data were analyzed from three experiments.

(c) Treatment with GW4869 decreased the levels of miRNAs in PN24 cell derived EVs/Exosomes compared to those derived from untreated control cells by qRT-PCR analysis, which was compared between the GW4869 treated and control cells with 1mg EVs/exosomes proteins. Data were analyzed from three experiments.

All statistical data are represented as mean ± SEM, and p-values are calculated by unpaired Student’s t-test.

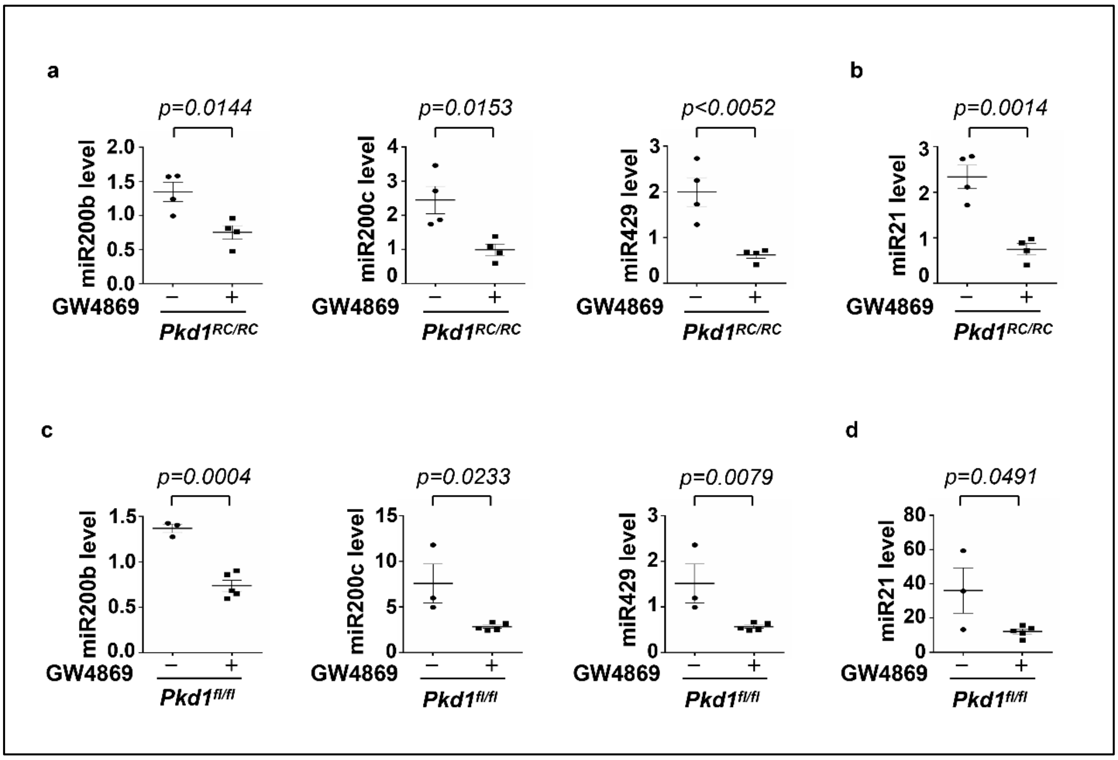

Supplementary Figure 12. Treatment with GW4869 decreased the expression of miRNAs miR200s and miR21 in kidneys from *Pkd1*$^{RC/RC}$ mice.

(**a** and **b**) qRT-PCR analysis of the expression of miR200b, miR200c, miR429 (**a**) and miR21 (**b**) in kidneys of *Pkd1*$^{RC/RC}$ mice treated with GW4869 or DMSO (n=4).

(**c** and **d**) qRT-PCR analysis of the expression of miR200b, miR200c and miR429 (**c**) as well as miR21 (**d**) in kidneys of *Pkd1*$^{flox/flox}$:*Pkhd1-Cre* mice treated with GW4869 or DMSO (n=3-5).

All statistical data are represented as mean ± SEM, and p-values are calculated by unpaired Student's t-test.

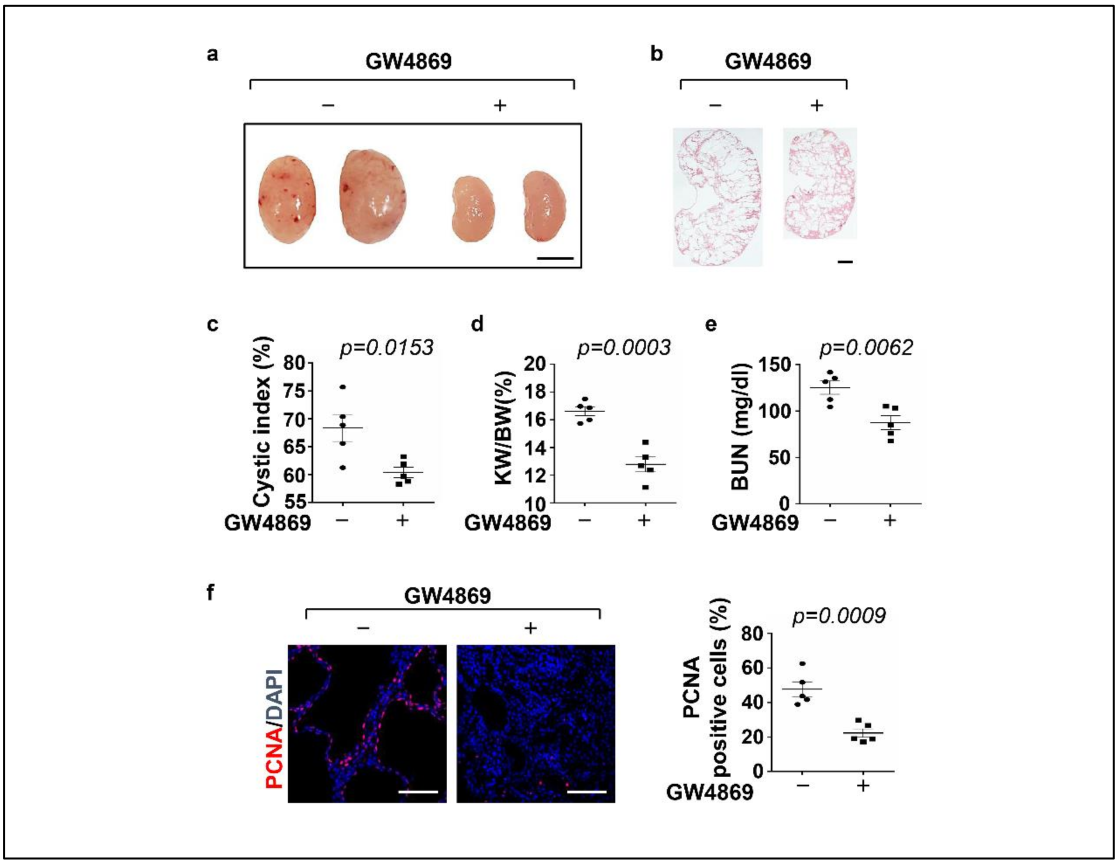

Supplementary Figure 13. Treatment with GW4869 delayed cyst growth in *Pkd1^flox/flox^:Pkhd1-Cre* mice.

(**a**) Images of kidneys from *Pkd1*$^{flox/flox}$:*Pkhd1-Cre* mice treated with or without GW4869. Scale bars, 5 mm.

(**b**) Histological examination of kidneys from *Pkd1*$^{flox/flox}$:*Pkhd1-Cre* mice treated with or without GW4869. Scale bars, 1 mm.

(**c**) Cyst index of kidneys from *Pkd1*$^{RC/RC}$ mice treated with GW4869 (n = 10) or DMSO (n = 5).

(**d** and **e**) Treatment with GW4869 decreased KW/BW ratios (**d**) and BUN levels (**e**) in *Pkd1*$^{flox/flox}$:*Pkhd1-Cre* mice compared to those in control mice with DMSO (n=5).

(**f**) Treatment with GW4869 reduced cyst-lining and interstitial cell proliferation in kidneys from *Pkd1*$^{flox/flox}$:*Pkhd1-Cre* mice as detected with PCNA staining. Scale bars, 100 μm. The percentage of PCNA positive cells was calculated from an average of 1,000 nuclei per mouse kidney section (n=5).

All statistical data are represented as mean ± SEM, and p-values are calculated by unpaired Student’s t-test.

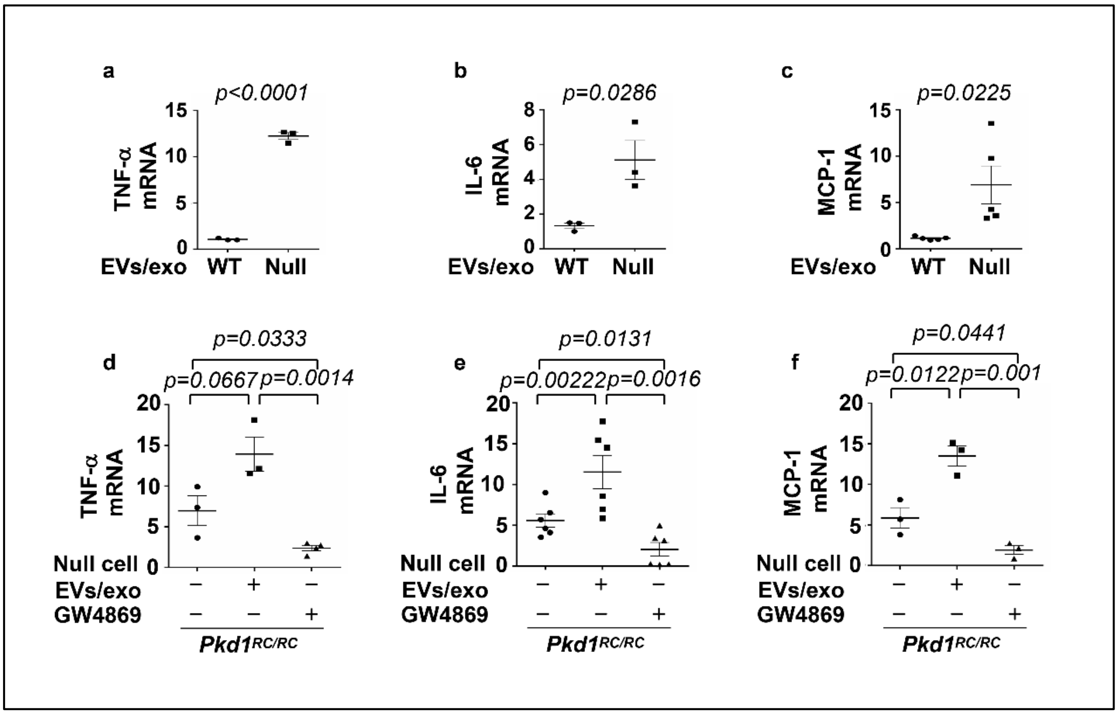

Supplementary Figure 14. Treatment with *Pkd1*-null cell EVs/exosomes increased the expression of TNF-α, IL-6 and MCP-1 in renal epithelial cells and tissues.

(**a**-**c**) qRT-PCR analysis of the expression of TNF-α (**a**), IL-6 (**b**) and MCP-1(**c**) in mIMCD3 cells treated with wild type cell EVs/exosomes or *Pkd1*-null cell EVs/exosomes. Data were analyzed from 3-5 experiments.(**d**-**f**) qRT-PCR analysis of the expression of TNF-α (**d**), IL-6 (**e**) and MCP-1(**f**) in kidneys of $Pkd1^{RC/RC}$ mice treated with *Pkd1*-null cell EVs/exosomes or GW4869. Data were analyzed from 3-6 experiments.

All statistical data are represented as mean ± SEM in **a**-**f**, and p-values are calculated by one-way ANOVA followed by Tukey's post hoc test in **d**-**f**, and by two-tailed unpaired t tests in **a**-**c**.

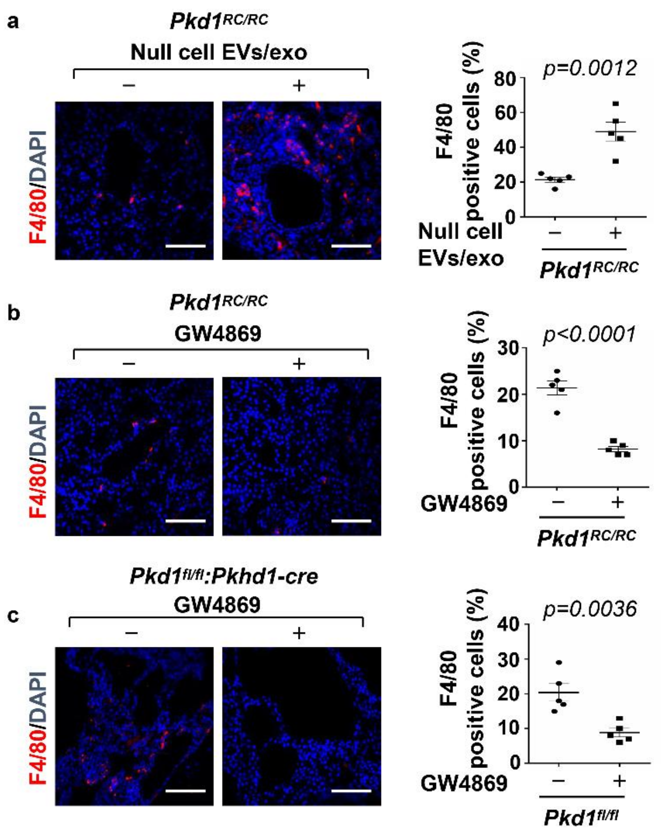

Supplementary Figure 15. Treatment with *Pkd1*-null cell EVs/exosomes and GW4869 affected the recruitment of macrophages in cystic kidneys.

(**a**) F4/80 staining of kidneys from *Pkd1*$^{RC/RC}$ mice treated with or without *Pkd1*-null cell EVs/exosomes.

(**b**) F4/80 staining of kidneys from *Pkd1*$^{RC/RC}$ mice treated with GW4869 or DMSO.

(**c**) F4/80 staining of kidneys from *Pkd1*$^{flox/flox}$*:Pkhd1-Cre* mice treated with GW4869 or DMSO.

n = 5 independent experiments. All statistical data are represented as mean ± SEM, and p-values are calculated by unpaired Student's t-test.

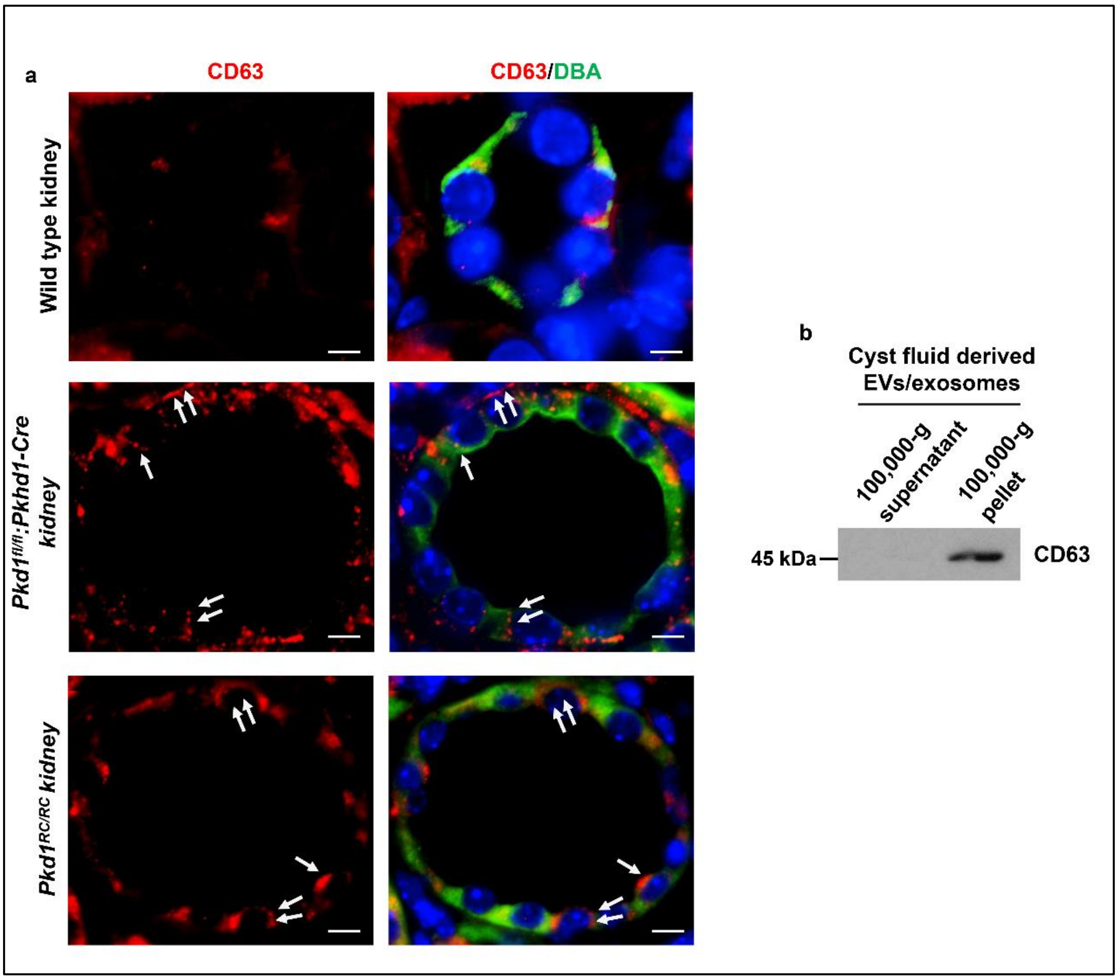

Supplementary Figure 16. The localization of EVs/exosomes in *Pkd1* mutant kidneys and EVs/exosomes in ADPKD cysts.

(**a**) Representative images of kidneys from wild type, *Pkd1$^{flox/flox}$:Pkhd1-Cre* and *Pkd1$^{RC/RC}$* mice stained with CD63, indicating an apical and basolateral localization of EVs/exosomes on normal tubular and cyst lining epithelial cells (*arrows*).

(**b**) EVs/Exosomes were isolated from cyst fluid of ADPKD patients by sequential centrifugation and was detected by Western blot analysis with the CD63 antibody.

Supplementary table 1. Primers used for quantitative real time PCR.

**Gene name**	**Forward (5'-3')**	**Reverse (5'-3')**
Actin	AAGAGCTATGAGCTGCCTGA	TACGGATGTCAACGTCACAC
Pkd1	CCCCGAATGTGGTTTCTATGG	GCCGTCCGATGTATGACTGC
PCNA	GGGTGAAGTTTTCTGCAAGTG	GTACCTCAGAGCAAACGTTAG G
Fibronectin	CTTTGGCAGTGGTCATTTCAG	ATTCTCCCTTTCCATTCCCG
α-SMA	GTCCCAGACATCAGGGAGTAA	TCGGATACTTCAGCGTCAGGA
TGF-β1	CTGCTGACCCCCACTGATAC	AGCCCTGTATTCCGTCTCCT
Collagen1	GCTCCTCTTAGGGGCCAC	CCACGTCTCACCATTGGGG
miR200b-3p	CGC AGT AAT ACT GCC TGG T	GGT CCA GTT TTT TTT TTT TTT TCA TCA
miR200c-3p	AGT AAT ACT GCC GGG TAA TGA	GGT CCA GTT TTT TTT TTT TTT TCC A
miR429-3p	GCA GTA ATA CTG TCT GGT AAT GC	GGT CCA GTT TTT TTT TTT TTT TAC G
miR21-5p	GCA GTA GCT TAT CAG ACT GAT G	GGT CCA GTT TTT TTT TTT TTT TCA AC
U6	CGCTTCGGCAGCACATATAC	CAGGGGCCATGCTAATCTT
nSMase2	TTTACTCGCTACAAAGACCCC	CTTCCTCGCTCTCCAACAC
Rab27a	CGACCTGACAAATGAGCAAAG	CCTCTTTCACTGCCCTCTG
TNF- α	ACCCTCACACTCAGATCATCTTC	TGGTGGTTTGCTACGACGT
IL-6	TAGTCCTTCCTACCCCAATTTCC	TTGGTCCTTAGCCACTCCTTC
MCP-1	TCTGGGCCTGCTGTTCACA	GGATCATCTTGCTGGTGAATG A
