# Supplementary material for: Extracellular vesicles and exosomes generated from cystic renal epithelial cells promote cyst growth in autosomal dominant polycystic kidney disease
Source: Nat Commun. 2021 Jul 27;12:4548. doi: 10.1038/s41467-021-24799-x (PMC8316472; doi:10.1038/s41467-021-24799-x)
Supplement: Supplementary file 2 — Reporting Summary [file 41467_2021_24799_MOESM2_ESM.pdf]

nature portfolio

Corresponding author(s): Xiaogang Li

Last updated by author(s): Jun 30, 2021

Reporting Summary

Nature Portfolio wishes to improve the reproducibility of the work that we publish. This form provides structure for consistency and transparency in reporting. For further information on Nature Portfolio policies, see our Editorial Policies and the Editorial Policy Checklist.

Statistics

For all statistical analyses, confirm that the following items are present in the figure legend, table legend, main text, or Methods section.

n/a	Confirmed	
☐	☒	The exact sample size (*n*) for each experimental group/condition, given as a discrete number and unit of measurement
☐	☒	A statement on whether measurements were taken from distinct samples or whether the same sample was measured repeatedly
☐	☒	The statistical test(s) used AND whether they are one- or two-sided<br>*Only common tests should be described solely by name; describe more complex techniques in the Methods section.*
☒	☐	A description of all covariates tested
☒	☐	A description of any assumptions or corrections, such as tests of normality and adjustment for multiple comparisons
☐	☒	A full description of the statistical parameters including central tendency (e.g. means) or other basic estimates (e.g. regression coefficient) AND variation (e.g. standard deviation) or associated estimates of uncertainty (e.g. confidence intervals)
☐	☒	For null hypothesis testing, the test statistic (e.g. *F*, *t*, *r*) with confidence intervals, effect sizes, degrees of freedom and *P* value noted<br>*Give P values as exact values whenever suitable.*
☒	☐	For Bayesian analysis, information on the choice of priors and Markov chain Monte Carlo settings
☒	☐	For hierarchical and complex designs, identification of the appropriate level for tests and full reporting of outcomes
☒	☐	Estimates of effect sizes (e.g. Cohen's *d*, Pearson's *r*), indicating how they were calculated

Our web collection on statistics for biologists contains articles on many of the points above.

Software and code

Policy information about availability of computer code

Data collection	Carl Zeiss Microscopy Gmbh, Jena, Germany<br>Bruker AVANCE Ⅲ-700 （16.4 T） Vertical-bore two channel multinuclear spectrometer
Data analysis	Microsoft Exel 2010, v14.0.6023.10000 (64 Bit)<br>SPSS Statistics 22 software<br>Image-Pro Plus V5 software<br>Analyze 12.0 software<br>Adobe Photoshop CS 6.0

For manuscripts utilizing custom algorithms or software that are central to the research but not yet described in published literature, software must be made available to editors and reviewers. We strongly encourage code deposition in a community repository (e.g. GitHub). See the Nature Portfolio guidelines for submitting code & software for further information.

Data

Policy information about availability of data

All manuscripts must include a data availability statement. This statement should provide the following information, where applicable:

- Accession codes, unique identifiers, or web links for publicly available datasets
- A description of any restrictions on data availability
- For clinical datasets or third party data, please ensure that the statement adheres to our policy

Source data are provided as a Source Data file.
The authors declare that the data supporting the findings of this study are available within the paper and its supplementary information files.

Field-specific reporting

Please select the one below that is the best fit for your research. If you are not sure, read the appropriate sections before making your selection.

☒ Life sciences ☐ Behavioural & social sciences ☐ Ecological, evolutionary & environmental sciences

For a reference copy of the document with all sections, see nature.com/documents/nr-reporting-summary-flat.pdf

Life sciences study design

All studies must disclose on these points even when the disclosure is negative.

Sample size	The sample size were 5 animals per group. These sample sizes were thought to be statistically sufficient based on literatures involving PKD mouse models. P values were calculated by 2-tailed unpaired Student's t-test and 1-way ANOVA, and a P value less than 0.05 was considered significant.
Data exclusions	No data exclusion
Replication	Experiments were performed independently at least 3 times
Randomization	Random
Blinding	The investigators were blinded to group allocation during data collection and/or analysis

Reporting for specific materials, systems and methods

We require information from authors about some types of materials, experimental systems and methods used in many studies. Here, indicate whether each material, system or method listed is relevant to your study. If you are not sure if a list item applies to your research, read the appropriate section before selecting a response.

Materials & experimental systems

n/a	Involved in the study	
☐	☒	Antibodies
☐	☒	Eukaryotic cell lines
☒	☐	Palaeontology and archaeology
☐	☒	Animals and other organisms
☐	☒	Human research participants
☒	☐	Clinical data
☒	☐	Dual use research of concern

Methods

n/a	Involved in the study	
☒	☐	ChIP-seq
☒	☐	Flow cytometry
☒	☐	MRI-based neuroimaging

Antibodies

Antibodies used	The antibodies used for Western blot analysis included (a) anti-Rab27a (sc-74586), nSMase2 (sc-166637), anti-PCNA (sc25280), anti-fibronectin (sc-59826), which were purchased from Santa Cruz Biotechnology Inc.; (b) anti-[illegible]-SMA (ab7817), anti-collagen 1 (ab34710), anti-TSG101 (ab125011), anti-ALIX (ab 275377) and CD63 (ab217345). which were purchased from abcam; (c) anti-STAT3 (no. 9139), anti-ERK (no. 4696), anti-S6 (no. 2217), anti-Rb (no. 9309), anti-AKT (no. 9272), anti-4EBP-1(no. 9644), and the phosphorylated antibodies for STAT3-Y705 (no. 9131), ERK-T202/Y204 (no. 9101), S6-S235/236 (no. 2211), AKT-S473 (no. 9271), 4EBP-1-S65 (no. 9451) and Rb-S780 (no. 9307), which were purchased from Cell Signaling Technology; and (d) anti-actin antibody (A2228) and anti-tubulin (T7941) antibody, which were purchased from Sigma-Aldrich. In addition, the anti-PC1 antibody (7e12) was generated by Mayo Clinic 69. The secondary antibodies, including donkey anti-rabbit IgG–horseradish peroxidase (sc-2313), donkey anti-goat IgG–horseradish peroxidase (sc-2020), and goat anti-mouse IgG–horseradish peroxidase (sc-2005), were purchased from Santa Cruz Biotechnology Inc.
Validation	Western blot internal control antibodies Tubulin and B-actin are routinely used in our lab and have been validated using a variety of mammalian cell lines and tissues. A specific band at the expected molecular weight is observed with low background noise to signal ratio. The primary antibodies used were chosen based on already published data with the relevant citations included in the manuscript.<br>For staining, a monoclonal mouse anti-nSMase 2 antibody (1:50 dilution) and Rab27a (1:50 dilution), biotinylated secondary antibody (1: 100 dilution), and DAB substrate system were used.<br>Macrophages were detected by immunofluorescence staining (IF staining) with a pan-macrophage marker, F4/80. After antigen retrieval, tissue sections were incubated with a rat anti-mouse F4/80 antibody (14-4801-82; eBioscience Inc.; 1:100 dilution) overnight, and then were incubated with Fluro-555 anti-rat IgG secondary antibody and mounted in Prolong Gold Antifade reagent with DAPI (Invitrogen).<br>For Western blot analysis, the primary antibodies (1:1000 dilution) were used.

Eukaryotic cell lines

Policy information about cell lines

Cell line source(s)	All cells used were purchased from ATCC except PH2 and PN24 through the George M O'Brien Kidney Center at Yale University (NIH P30 DK079310).
Authentication	Cell lines were not authenticated
Mycoplasma contamination	All cells were not tested for mycoplasma due to all cell lines were immortalize cell line. Cell were maintained at a low passage.
Commonly misidentified lines (See ICLAC register)	There is no commonly misidentified cell lines were used in the study.

Animals and other organisms

Policy information about studies involving animals; ARRIVE guidelines recommended for reporting animal research

Laboratory animals	Both male and female Pkd1RC/RC mice (1 month to 3month old) and Pkd1 flox/flox:Pkhd1-Cre mice (7 to 28 days old mice) were randomly used in this study.<br>A 14-hour light/10-hour dark cycle or 12 light/12 dark cycle was used with temperatures of 65-75°F and 40-60% humidity. Ensure lights are not used and that researchers and technicians do not enter the mouse room during the dark cycle.
Wild animals	This study did not involve wild animals.
Field-collected samples	This study did not involve field collected animals.
Ethics oversight	All experiments involving animals were conducted under the approval of Mayo Clinic IACUC.

Note that full information on the approval of the study protocol must also be provided in the manuscript.

Human research participants

Policy information about studies involving human research participants

Population characteristics	Approximately 100 ml of first-void urines were collected from 5 healthy human volunteers and 5 ADPKD patients between the ages of 18 and 40 years with an estimated glomerular filtration rate (eGFR) between 40 and 80 mL/min/1.73 m2, evaluated by the abbreviated Modification of Diet in Renal Disease (MDRD) formula.
Recruitment	All participants were recruited randomly with no bias.
Ethics oversight	We confirmed that our study is compliant with the "Guidance of the Ministry of Science and Technology (MOST) for the Review and Approval of Human Genetic Resources".

Note that full information on the approval of the study protocol must also be provided in the manuscript.
